# Induction of Oral Immune Tolerance Enables Sequential AAV9/AAV8 Gene Therapy in Mucopolysaccharidosis IVA

**DOI:** 10.3390/ijms27219553

**Published:** 2026-10-26

**Authors:** Sampurna Saikia, Krishna Sai Musini, Shaukat Khan, Shunji Tomatsu

**Affiliations:** 1 Department of Pediatrics, Oregon Health and Science University, Portland, OR 97239, USA; saikia@ohsu.edu; 2 Department of Biomedical Research, Nemours Children’s Health, Wilmington, DE 19803, USA; krishnasai.musini@nemours.org (K.S.M.); shaukat.khan@nemours.org (S.K.); 3 Department of Pediatrics, Shimane University, Izumo 693-8501, Japan; 4 Department of Pediatrics, Graduate School of Medicine, Gifu University, Gifu 501-1193, Japan; 5 Department of Pediatrics, Thomas Jefferson University, Philadelphia, PA 19107, USA; 6 Department of Biological Sciences, University of Delaware, Newark, DE 19716, USA; 7 DuPont Experimental Station, Bldg. E400 #5205, 200 Powder Mill Rd., Wilmington, DE 19803, USA

**Keywords:** immune tolerance, Morquio A syndrome, AAV gene therapy, humoral immune response, cytotoxic immune response, AAV serotype

## Abstract

Mucopolysaccharidosis IVA (MPS IVA) results from deficiency of N-acetylgalactosamine-6-sulfate sulfatase (GALNS), leading to progressive keratan sulfate accumulation, and current treatments have limited efficacy. Although adeno-associated virus (AAV) gene therapy can restore systemic enzyme activity, immune responses to the transgene and viral capsid restrict durability and prevent vector re-administration. We evaluated a combined strategy of antigen-specific oral immune tolerance and sequential AAV serotype delivery. Neonatal MPS IVA mice received a mixture of three immunodominant GALNS-derived T- and B-cell epitopes on alternate days for 20 days, followed by AAV9 at 4 weeks and redosing with either AAV9 or AAV8 at 10 weeks. Peptide-treated tolerized MPS IVA mice showed a 2–3-fold increase in circulating GALNS activity after the first dose and a 7–8-fold increase after redosing, whereas non-tolerized MPS IVA mice developed anti-GALNS antibodies that reduced enzyme levels. No cross-reactive neutralizing antibodies were detected between AAV9 and AAV8. Peptide-treated mice showed normalization of plasma and hepatic keratan sulfate (KS) and significant improvements in skeletal and cardiac histopathology, with the greatest benefit in the peptide-treated AAV9→AAV8 group. These findings demonstrate that oral immune tolerance enables safe AAV re-administration, enhances systemic enzyme bioavailability, and improves tissue correction, supporting a clinically translatable approach for durable treatment of MPS IVA.

## 1. Introduction

Mucopolysaccharidosis IVA (MPS IVA; Morquio A syndrome) is an autosomal recessive lysosomal storage disorder caused by pathogenic variants in the *GALNS* gene, which encodes N-acetylgalactosamine-6-sulfate sulfatase (GALNS). Loss of GALNS activity impairs degradation of keratan sulfate (KS) and chondroitin-6-sulfate (C6S), leading to progressive glycosaminoglycan (GAG) accumulation within chondrocytes and connective tissues [1]. Clinically, MPS IVA is characterized by short stature, severe skeletal dysplasia, joint hypermobility, airway obstruction, corneal clouding, and restrictive lung disease [2]. Unlike other MPS subtypes, Morquio A predominantly affects the skeletal system, posing unique therapeutic challenges because cartilage is avascular and connective tissues have high metabolic demands.

Current clinical management relies primarily on enzyme replacement therapy (ERT). Although ERT reduces the systemic GAG burden and modestly improves endurance, its impact on skeletal pathology is limited because intravenously administered enzyme poorly penetrates cartilage and requires lifelong weekly infusions. Moreover, ERT frequently induces anti-drug antibodies and T-cell-mediated immune responses, thereby accelerating enzyme clearance and diminishing efficacy. Hematopoietic stem cell transplantation (HSCT) has also been investigated; however, its clinical utility remains constrained by donor availability, graft-versus-host disease, graft failure, and minimal benefit for skeletal disease [3,4]. Collectively, these limitations highlight the need for durable, tissue-penetrant, and less immunogenic therapeutic approaches.

Adeno-associated virus (AAV) mediated gene therapy has emerged as a promising strategy for lysosomal storage disorders. Preclinical studies across multiple MPS models show that hepatocyte-directed AAV delivery can achieve sustained enzyme expression, reduce GAG accumulation, and improve functional outcomes [1]. In MPS IVA, AAV-mediated GALNS expression can provide a continuous systemic enzyme supply and broader tissue distribution than ERT [5]. However, immune responses against the GALNS transgene, capsid-specific neutralizing antibodies (NAbs), and cytotoxic T lymphocyte (CTL) responses against transduced cells remain major barriers to durable efficacy [6].

High-dose AAV administration has been associated with serious adverse events, including glomerulonephritis resulting from immune complex deposition in the kidneys, underscoring the narrow safety margin of single-dose approaches [7,8]. Notably, conventional immunosuppressive regimens have not reliably mitigated these risks, supporting a therapeutic paradigm based on repeated administration of moderate AAV doses [9]. However, AAV re-administration is limited by the rapid induction of serotype specific NAbs following initial exposure, which can persist long-term and block subsequent transduction by the same serotype [7,9,10].

Although AAV vectors are often expected to mediate durable transgene expression, redosing may be required because of hepatocyte turnover, vector genome dilution during pediatric liver growth, or insufficient initial expression [11,12,13]. Therefore, strategies that attenuate immune responses to both the transgene and the AAV capsid are essential to enable safe and effective repeated dosing at clinically feasible vector doses.

Antigen-specific immune tolerance approaches have gained increasing attention as adjuncts to gene therapy. Oral tolerance induction, achieved through repeated oral exposure to antigen-derived peptides, promotes regulatory immune responses within gut-associated lymphoid tissue, resulting in T cell anergy, deletion, or the induction of regulatory T cells [14]. This strategy has demonstrated efficacy in preclinical models of hemophilia, rheumatoid arthritis, and other allergy- or autoimmune-related diseases by suppressing anti-drug antibody formation and T-cell activation [15,16,17,18,19]. Importantly, oral tolerance is noninvasive, antigen-specific, and scalable, making it well-suited for combination with gene therapies that require sustained transgene expression [20,21,22]. We have successfully induced oral immune tolerance using GALNS-derived peptide epitopes administered to neonatal mice before AAV9-mediated gene delivery [23]. In a non-inflammatory context, the immunodominant hGALNS epitopes previously used in this study were identified and validated for murine MHC II binding using established prediction platforms such as IEDB and RANKPEP [24]. These methodologies also allow the prediction of peptide binding to human MHC II molecules. We selected three immunodominant epitopes identified by Sosa et al. that elicited the strongest CD4^+^ T-cell responses [24]. In the context of tolerogenic delivery, these epitopes enable strong antigen engagement and induce antigen-specific immune tolerance.

Building on this work, the present study addresses AAV capsid immunogenicity by integrating oral peptide-based immune tolerance with dual-serotype AAV gene therapy using AAV9 and AAV8 vectors. Both serotypes exhibit strong liver tropism and support robust systemic secretion of lysosomal enzymes, enabling cross-correction of peripheral tissues [25]. Importantly, their distinct serological profiles enable a serotype-switching (“cross-administration”) strategy to circumvent NAb-mediated inhibition of vector re-administration [10]. Sequential administration of serologically distinct AAV serotypes has been shown in murine and non-human primate models to preserve transgene expression, whereas homologous redosing results in high NAb titers and loss of efficacy [26].

Based on these observations, we reasoned that a dual immunomodulatory strategy could overcome these barriers: antigen-specific oral immune tolerance to suppress GALNS-directed adaptive immunity and serotype switching to circumvent capsid-specific neutralization. Oral delivery of GALNS-derived peptides leverages physiological tolerance mechanisms within gut-associated lymphoid tissue, offering a noninvasive, scalable approach to restrict transgene-directed immune responses without systemic immunosuppression. Concurrently, sequential delivery of serologically distinct AAV serotypes with overlapping tissue tropism enables repeat vector administration while preserving transduction efficiency. Therefore, we hypothesized that coupling oral peptide-based immune tolerance with a dual serotype AAV delivery strategy would enable safe and effective AAV re-dosing, resulting in more durable GALNS expression and improved therapeutic outcomes in MPS IVA.

## 2. Results

**Figure 1:**
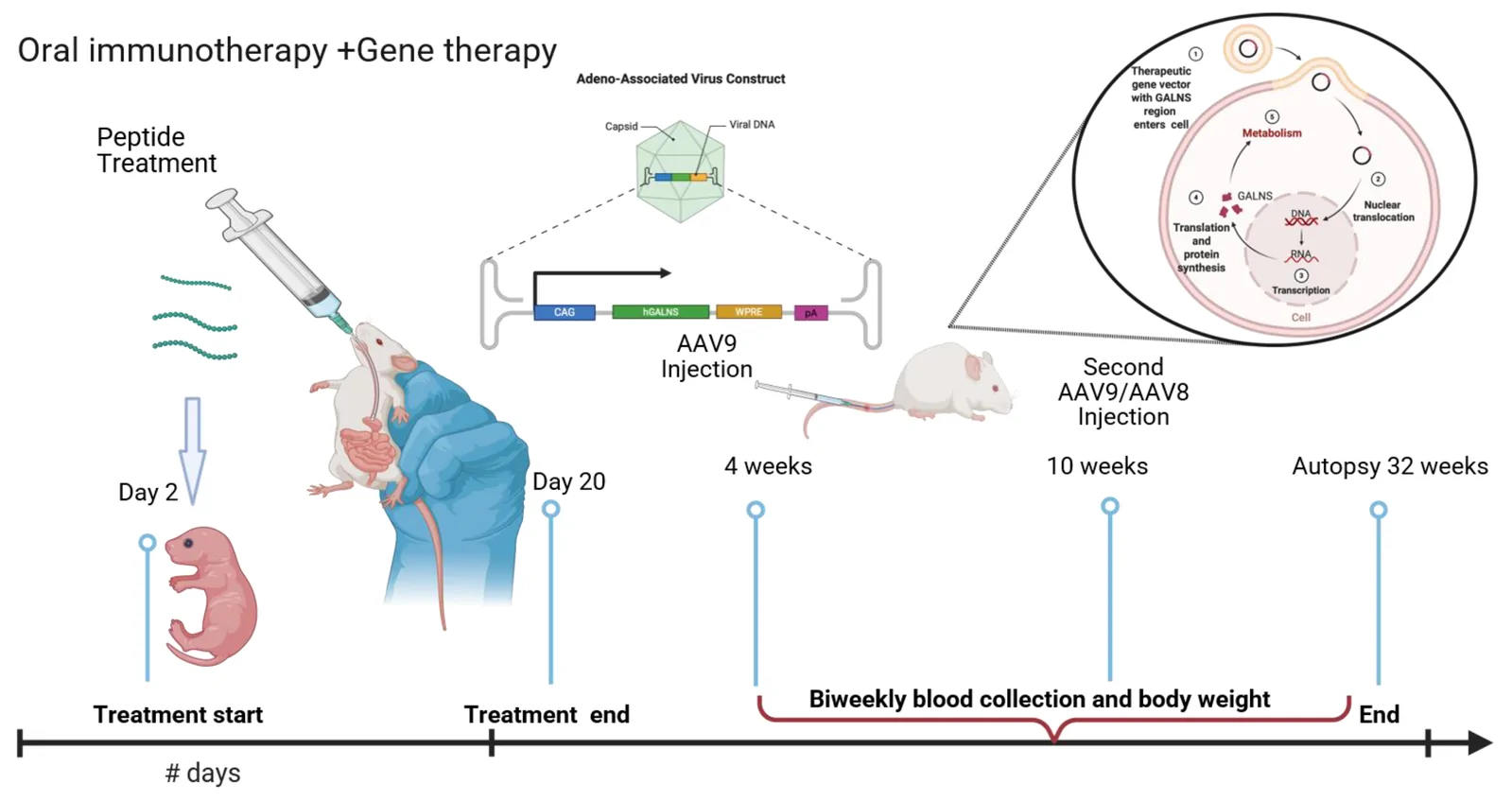
Schematic illustration of the treatment in the MPS IVA mouse model (GALNS^−/−^ KO).

In this study, we orally administered three immunodominant GALNS peptides to newborn *Galns* knockout (*Galns^−/−^*KO) mice, henceforth referred to as MPS IVA mice, and then injected them intravenously with AAV9 and AAV8 vectors encoding human GALNS (CAG-AAV9-hGALNS) (Figure 1, Supplementary Table S1). Untreated MPS IVA and wild-type mice served as controls.

### 2.1. Oral Peptide-Induced Immune Tolerance with Sequential AAV Delivery Enhances GALNS Activity

**Figure 2:**
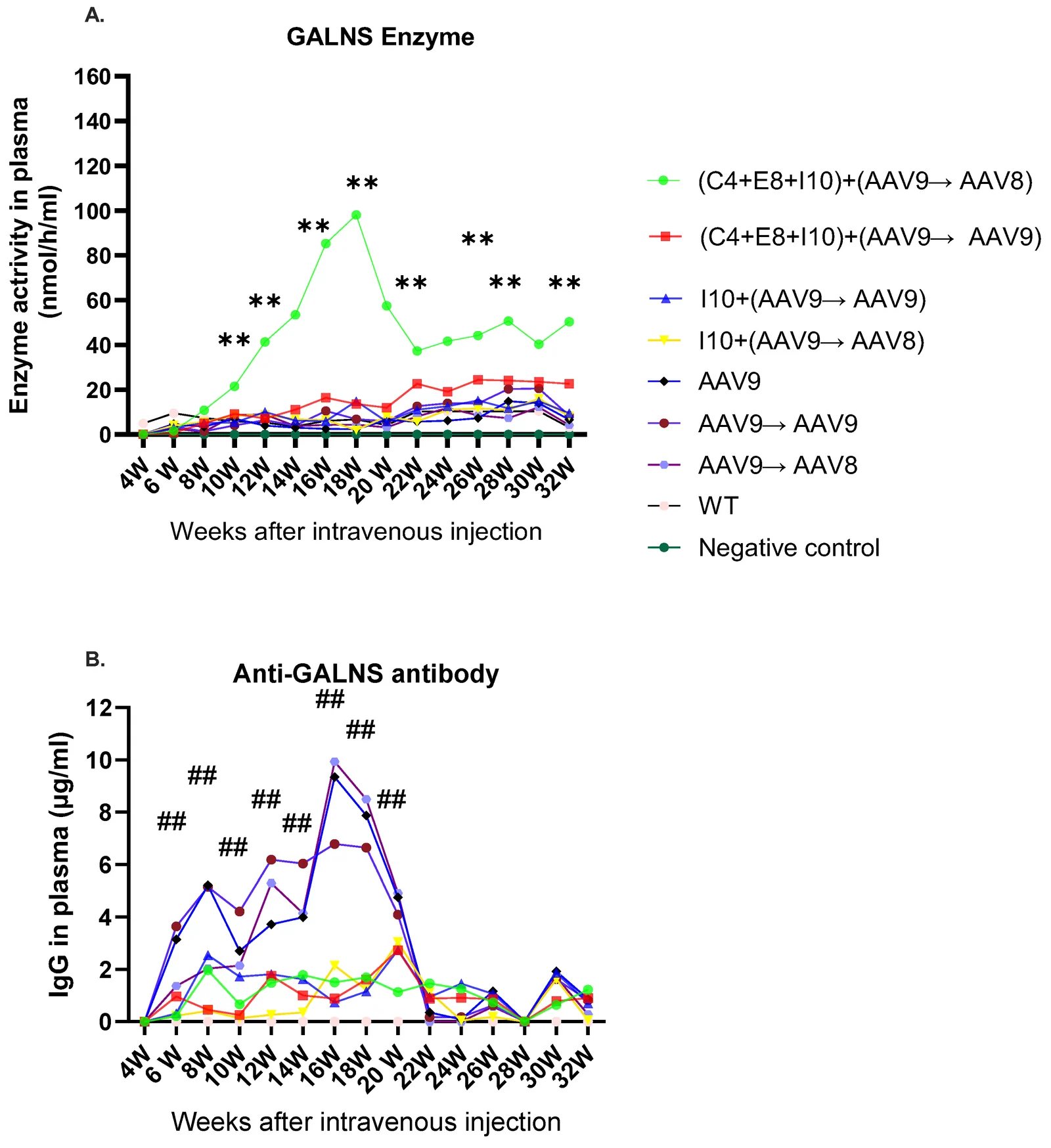
Levels of Biomarkers. (**A**) GALNS enzyme activity in plasma over time; *p*-values are expressed as **: <0.005, (statistical difference between [(C4 + E8 + I10) + (AAV9→AAV8)] with all pother treated groups). (**B**) Anti-GALNS IgG in plasma; *p*-values are expressed as ##: <0.005, (statistical difference between [(C4 + E8 + I10) + (AAV9→AAV8)] with all pother treated groups). One-way ANOVA with Tukey’s post hoc test, *n* = 3–11.

GALNS enzyme activities were quantified in plasma and tissues collected at the terminal time point of 32 weeks. Plasma enzyme activity increased by 4 weeks post-AAV gene therapy of 5 × 10^13^ genome copies/kg (AAV-GT) in all treated groups (*n* = 3–11). Among these, the immunotolerant group [(C4 + E8 + I10) + (AAV9→AAV8)] (Figure 2A) demonstrated the highest and most sustained enzyme activity throughout the study, exhibiting a >8-fold increase relative to untreated wild-type controls (38.85 nmol/h/mL). The [(C4 + E8 + I10) + (AAV9→AAV9)] group maintained stable enzyme activity through 32 weeks, corresponding to a 4-fold increase at the terminal point (22.63 nmol/h/mL). In contrast, the non-immunotolerant groups (AAV9, AAV9→AAV9, and AAV9→AAV8) displayed supraphysiological enzyme levels, though lower than those observed in the [(C4 + E8 + I10) + (AAV9→AAV8)] group. No significant differences in enzyme activity were detected among the I10 + (AAV9→AAV8), I10 + (AAV9→AAV9), AAV9, AAV9→AAV9, and AAV9→AAV8 groups across all time points.

**Figure 3:**
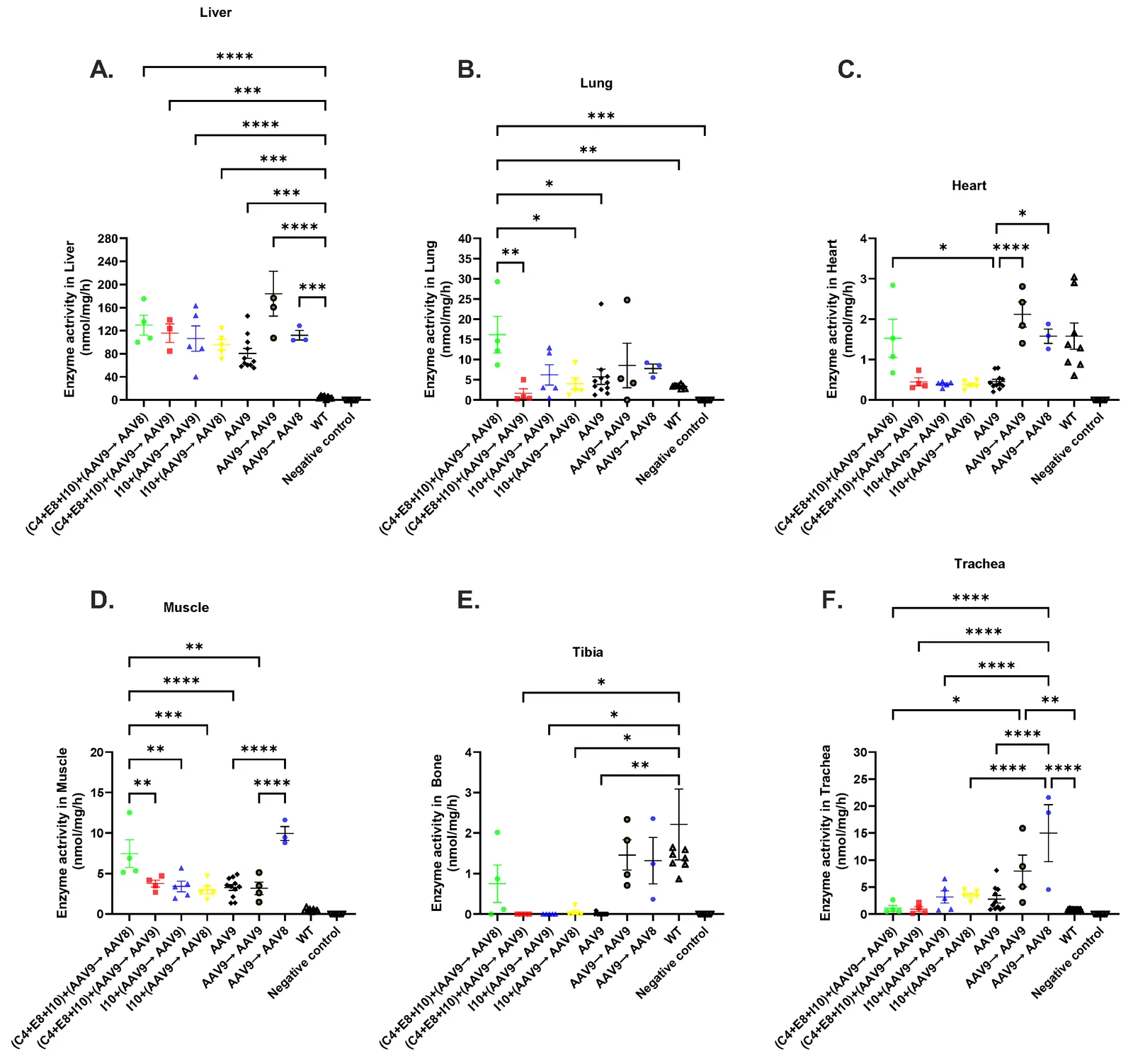
GALNS enzyme activity in different tissues after immune tolerance therapy and gene therapy at 32 weeks. (**A**) Liver; (**B**) Lung; (**C**) Heart; (**D**) Muscle; (**E**) Tibia; (**F**) Trachea. One-way ANOVA with Tukey’s post hoc test for plasma week-by-week; *: <0.05, **: <0.005, ***: <0.001, ****: <0.0001. *n* = 3–11. Error bars are for the standard error of the mean.

To assess cross-correction of GALNS deficiency, enzyme activities were measured in multiple tissues, including the liver, heart, lung, bone, muscle, and trachea, at 32 weeks post-treatment (Figure 3). Liver GALNS activity was significantly elevated in all treated groups compared with wild-type controls (Figure 3A). Notably, the [(C4 + E8 + I10) + (AAV9→AAV9)] and [(C4 + E8 + I10) + (AAV9→AAV8)] groups exhibited the highest liver enzyme activities, showing 35–40-fold increases relative to wild-type levels. A similar trend was observed in the non-immunotolerant AAV9→AAV9 and AAV9→AAV8 groups. In contrast, the single-dose AAV9-treated group demonstrated a more moderate yet significant 20-fold increase in liver enzyme activity (Figure 3A).

In the lung, the [(C4 + E8 + I10) + (AAV9→AAV8)] group exhibited the highest GALNS enzyme activity, which was significantly greater than that observed in all other treated and untreated groups. This group showed a 6-fold increase in enzyme activity relative to wild-type controls (Figure 3B).

In the heart, the [(C4 + E8 + I10) + (AAV9→AAV8)] group exhibited significantly higher GALNS enzyme activity than the single-dose AAV9 group. Similarly, both the dual-dose AAV9→AAV9 and AAV9→AAV8 groups showed significantly higher enzyme activity than the single-dose AAV9 group (Figure 3C).

In the muscle, the [(C4 + E8 + I10) + (AAV9→AAV8)] group exhibited the highest GALNS enzyme activity, which was significantly greater than that observed in all other treated and untreated groups, except for the non-immunotolerant AAV9→AAV8 group. Additionally, the AAV9→AAV8 group showed significantly higher enzyme activity than both the single-dose AAV9 and dual-dose AAV9→AAV9 groups (Figure 3D).

In the bone, only the AAV9→AAV9 and AAV9→AAV8 groups exhibited enzyme activities that were not significantly different from those of the wild-type controls. Among the immunotolerant groups, [(C4 + E8 + I10) +(AAV9→AAV8)] was the only group that showed detectable GALNS enzyme activity (Figure 3E). A similar trend was observed in the trachea, where the AAV9→AAV8 group had the highest GALNS enzyme activity, which was significantly greater than that of the immunotolerant groups and the single-dose AAV9 group. The [(C4 + E8 + I10) + (AAV9→AAV8)] group showed the second-highest enzyme activity among all treatment groups (Figure 3F) except trachea. By 32 weeks of age, antibody titers against both the GALNS transgene product and the AAV capsid were minimal, enabling efficient enzyme delivery to the tibia in the AAV9→AAV9 and AAV9→AAV8 groups. However, enzyme detection within the whole bone does not necessarily reflect effective delivery to the growth plate, a structurally and physiologically restricted compartment. All the groups showing statistical differences had GALNS activity similar to WT animals.

### 2.2. Peptide-Induced Immune Tolerance Suppresses Anti-GALNS Antibody Formation

To evaluate the immune response to GALNS protein produced after AAV GT, ELISA was performed to measure GALNS-specific antibody levels, using plasma collected biweekly until 32 weeks post-AAV-GT. We detected minimal total IgG antibodies against GALNS in the groups treated with peptides orally [(C4 + E8 + I10) + (AAV9→AAV8)] and [(C4 + E8 + I10) + (AAV9→AAV9)] throughout this period (Figure 2B). However, the groups receiving only one dose of AAV9, a double dose (AAV9→AAV9) or (AAV9→AAV8) with no oral tolerance induction showed elevated anti-GALNS IgG antibodies starting 2 weeks after AAV injection, peaking at 16 weeks, and gradually decreasing until 32 weeks. As expected, no antibody was detected in the untreated wild-type and MPS IVA groups. These findings suggest that immune tolerance was induced in groups that received orally administered immunodominant GALNS peptides. Antibody levels varied inversely with plasma enzyme activities (Figure 2A,B). The peptide groups, particularly the group with a double dose of GT of AAV8 and AAV9, exhibited the highest enzyme activity and the lowest levels of anti-GALNS IgG antibodies. Conversely, the only gene therapy-treated groups (AAV9), (AAV9→AAV8), and (AAV9→AAV9) without oral immune tolerance showed higher antibody levels and lower enzyme activity until 32 weeks. However, single-peptide orally administered groups [I10 + (AAV9→AAV9) and I10 + (AAV9→AAV8)] showed low enzyme activity similar to non-tolerized groups despite lower antibody levels, indicating ineffective immunotolerance.

Overall, these findings indicated that oral administration of GALNS-epitope peptides suppressed the anti-GALNS humoral response, as evidenced by the absence of antibody production.

### 2.3. Modulation of Cell-Mediated Immunity

**Figure 4:**
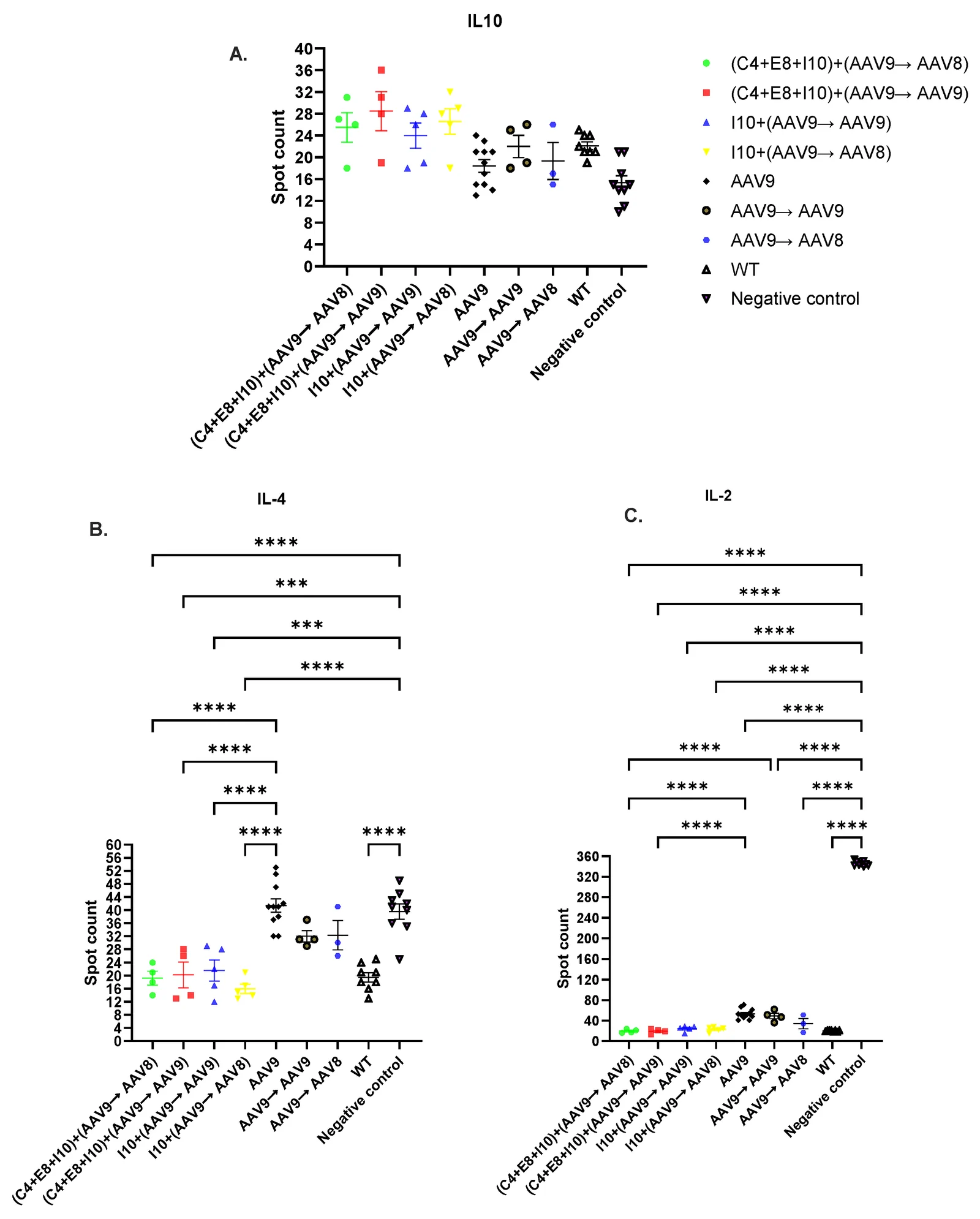
Cellular immune response in splenocytes at 32 weeks of age, measured by the number of cells producing (**A**) IL-10, (**B**) IL-2, and (**C**) IL-4. One-way ANOVA with Tukey’s post hoc test for plasma week-by-week; *p*-values are expressed as ***: <0.001, ****: <0.0001. *n* = 3–11. Error bars are for the standard error of the mean.

The immunosuppressive cytokine IL-10 is associated with oral immune tolerance in the gut-associated lymphoid tissue (GALT). To assess IL-10-secreting T cells, spleens were collected from mice at the 32-week endpoint, coinciding with the overall evaluation of AAV-GT following oral tolerance induction. Splenocytes were isolated and subjected to an IL-10 ELISPOT assay after stimulation with GALNS peptides. All immunotolerance induction groups exhibited higher numbers of IL-10-producing cells than the non-immunotolerant group (Figure 4A). Conversely, the number of T cells secreting the pro-inflammatory cytokines IL-2 and IL-4 was significantly reduced in immunotolerant groups (Figure 4B,C). Notably, IL-4 levels were significantly lower in the immunotolerant groups than in the AAV-only group. Together, these results suggest that peptide-induced immune tolerance modulates cell-mediated responses in a manner consistent with the observed humoral immune profiles.

### 2.4. Sequential AAV Administration Avoids Cross-Reactive Anti-Capsid Responses

**Figure 5:**
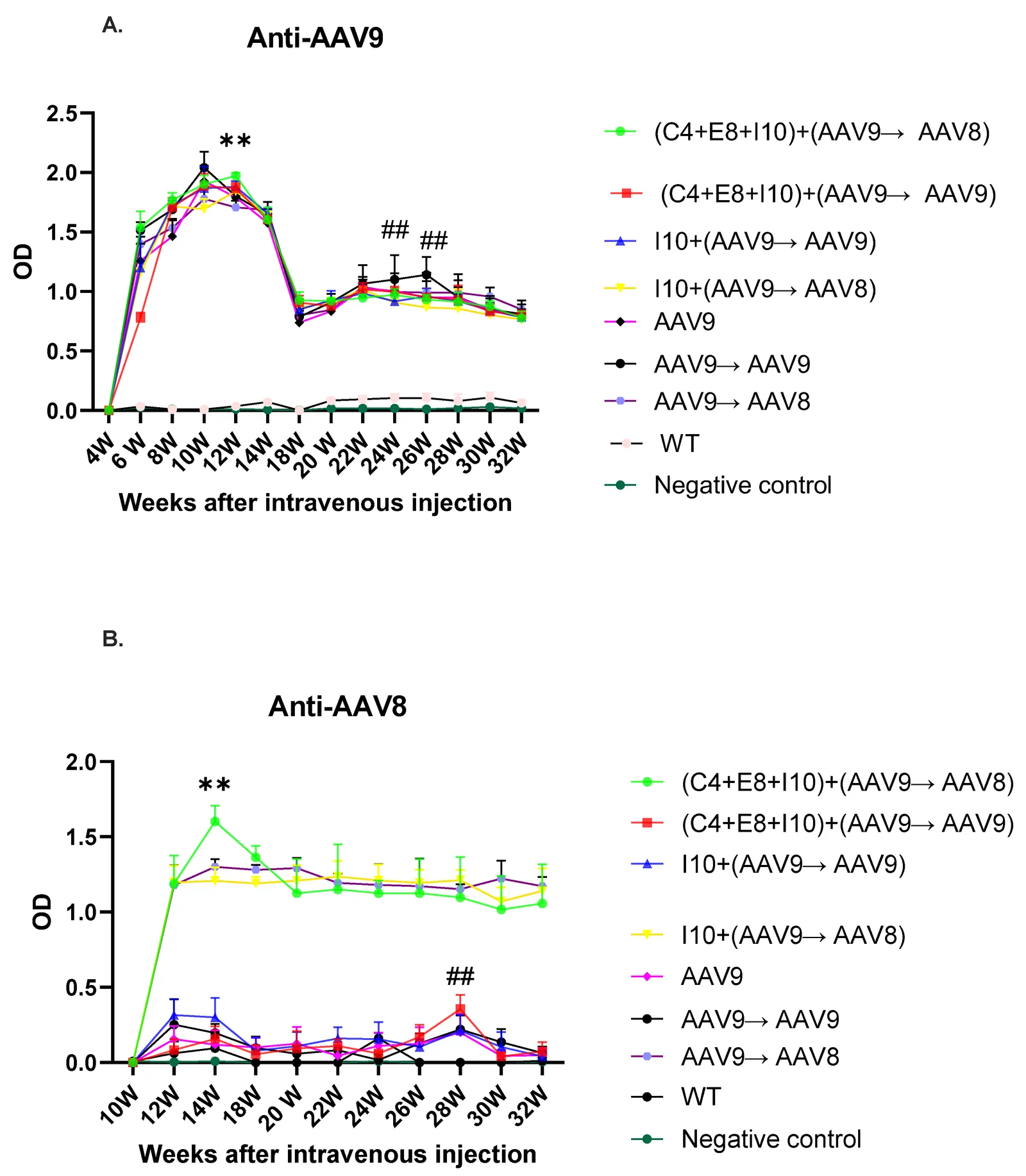
Anti-AAV IgG in plasma. (**A**) Anti-AAV9 (** = difference between [(C4 + E8 + I10) + (AAV9→AAV8)] and AAV9 group at 12 weeks; ## = at 24 and 28 weeks). (**B**) Anti-AAV8; *p*-values are expressed as **: <0.005 and ##: <0.005 (** = difference between [(C4 + E8 + I10) + (AAV9→AAV8)] and AAV9 group at 14 weeks; ## = all AAV8 treated groups vs. control groups). Two-way ANOVA with Tukey’s post hoc test, *n* = 3–11. Error bars are for the standard error of the mean.

To evaluate the immune response to AAV capsid proteins following AAV gene therapy (GT), capsid-specific antibodies were quantified by ELISA using plasma collected biweekly for up to 32 weeks post-AAV-GT. Moderate levels of total IgG antibodies against the AAV9 capsid were detected in all treated groups throughout the study period (Figure 5A). However, mice receiving a single dose of AAV9 or dual-dose regimens [(AAV9→AAV9) or (AAV9→AAV8)], with or without oral tolerance induction, exhibited reduced anti-AAV9 IgG levels from 18 to 32 weeks.

The single-dose AAV9 group had significantly lower anti-AAV9 IgG levels than the [(C4 + E8 + I10) + (AAV9→AAV8)] group at 12 weeks. In contrast, the dual-dose AAV9 group (AAV9→AAV9) had significantly higher antibody levels than all AAV9 + AAV8 groups, regardless of whether immune tolerance was induced. As expected, no anti-AAV antibodies were detected in untreated wild-type or MPS IVA mice.

For anti-AAV8 antibodies, all AAV8-treated groups had significantly higher IgG levels than wild-type, untreated, and non-AAV8-treated groups (Figure 5B). No significant differences were observed among AAV8-treated groups over time, except at 14 weeks, when the [(C4 + E8 + I10) + (AAV9→AAV8)] group had higher antibody levels. All non-AAV8-treated groups had antibody levels comparable to controls at all time points, except at 28 weeks, which may reflect nonspecific binding or background signal. After 32 weeks, AAV9 IgG levels had declined as the humoral immune response to the capsid naturally contracted. As long-lived plasma cells and immunological memory may maintain anti-AAV immunity even in the face of declining circulating IgG levels, this reduction should not be interpreted as proof of capsid-specific immune tolerance.

Overall, these results indicate that sequential administration of different AAV serotypes elicits limited cross-reactive antibodies, thereby supporting the enhanced efficacy of AAV-mediated gene therapy.

### 2.5. Biochemical Correction of Keratan Sulfate Storage

**Figure 6:**
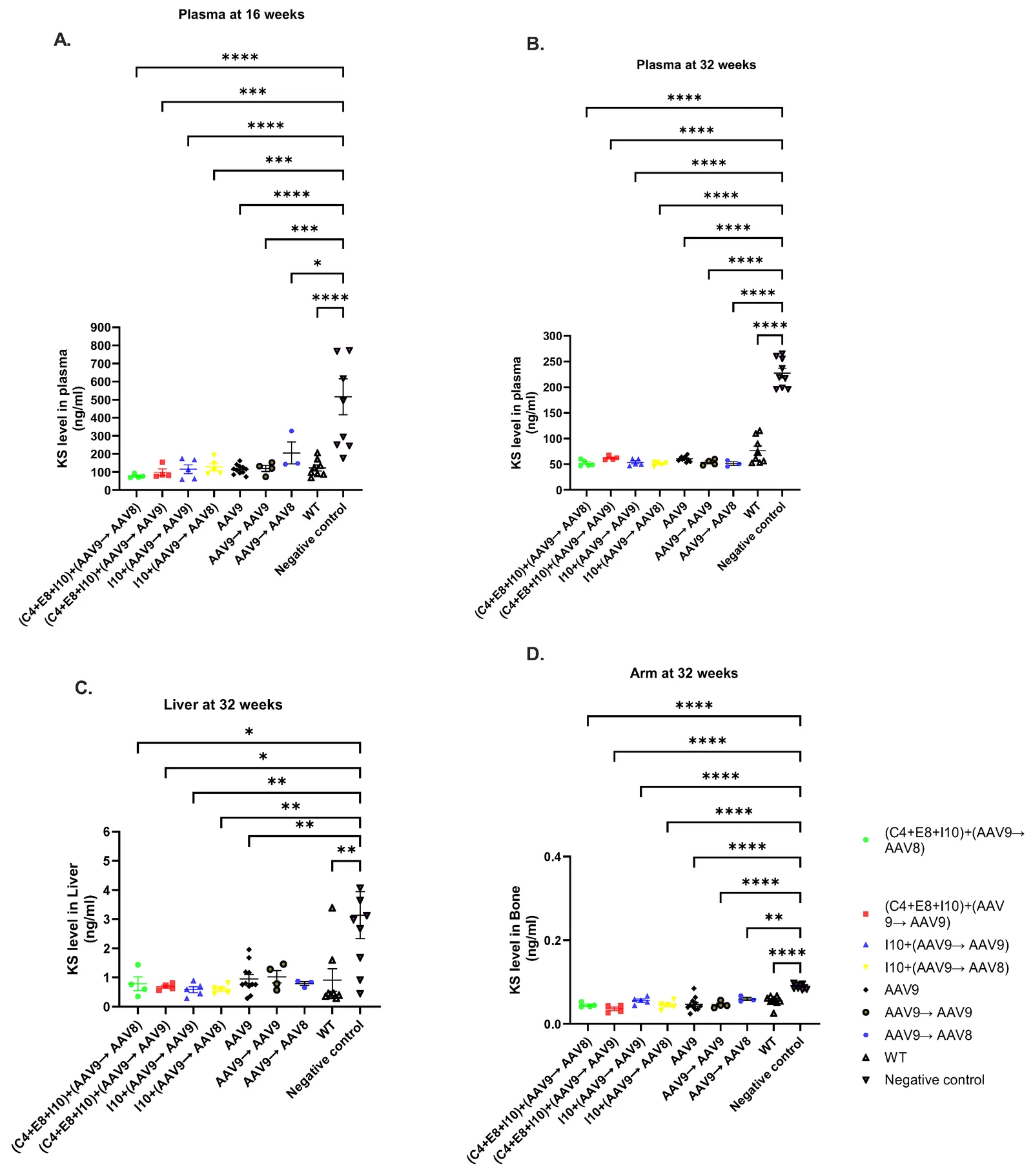
Mono-sulfated KS levels after immune tolerance therapy, followed by AAV gene therapy in MPS IVA mice. (**A**) Plasma at 16 weeks. (**B**) Plasma at 32 weeks. (**C**) Liver. (**D**) Bone. One-way ANOVA with Tukey’s post hoc test for plasma week-by-week; *: <0.05, **: <0.005, ***: <0.001, ****: <0.0001. *n* = 3–11. Error bars are for the standard error of the mean.

Levels of the primary storage material, mono-sulfated KS, were quantified in plasma at 16 and 32 weeks of age and in the liver and bone (arm) at 32 weeks (the time of autopsy) (Figure 6). Untreated MPS IVA mice exhibited significantly elevated KS levels in plasma, liver, and bone compared with all treated groups (Figure 6A–D). In contrast, all treated groups maintained plasma KS levels comparable to those of wild-type controls at both 16 and 32 weeks (Figure 6A,B). Similarly, KS levels in the liver and bone of treated mice were similar to those of wild-type mice, confirming effective correction of substrate accumulation (Figure 6C,D).

### 2.6. Histopathological Improvement in Skeletal and Cardiac Tissues

To assess improvement in pathological features, heart and knee-joint lesions were sectioned at 0.5 µm and stained with toluidine blue after autopsy at 32 weeks of age. Pathological improvement was then assessed [1].

**Figure 7:**
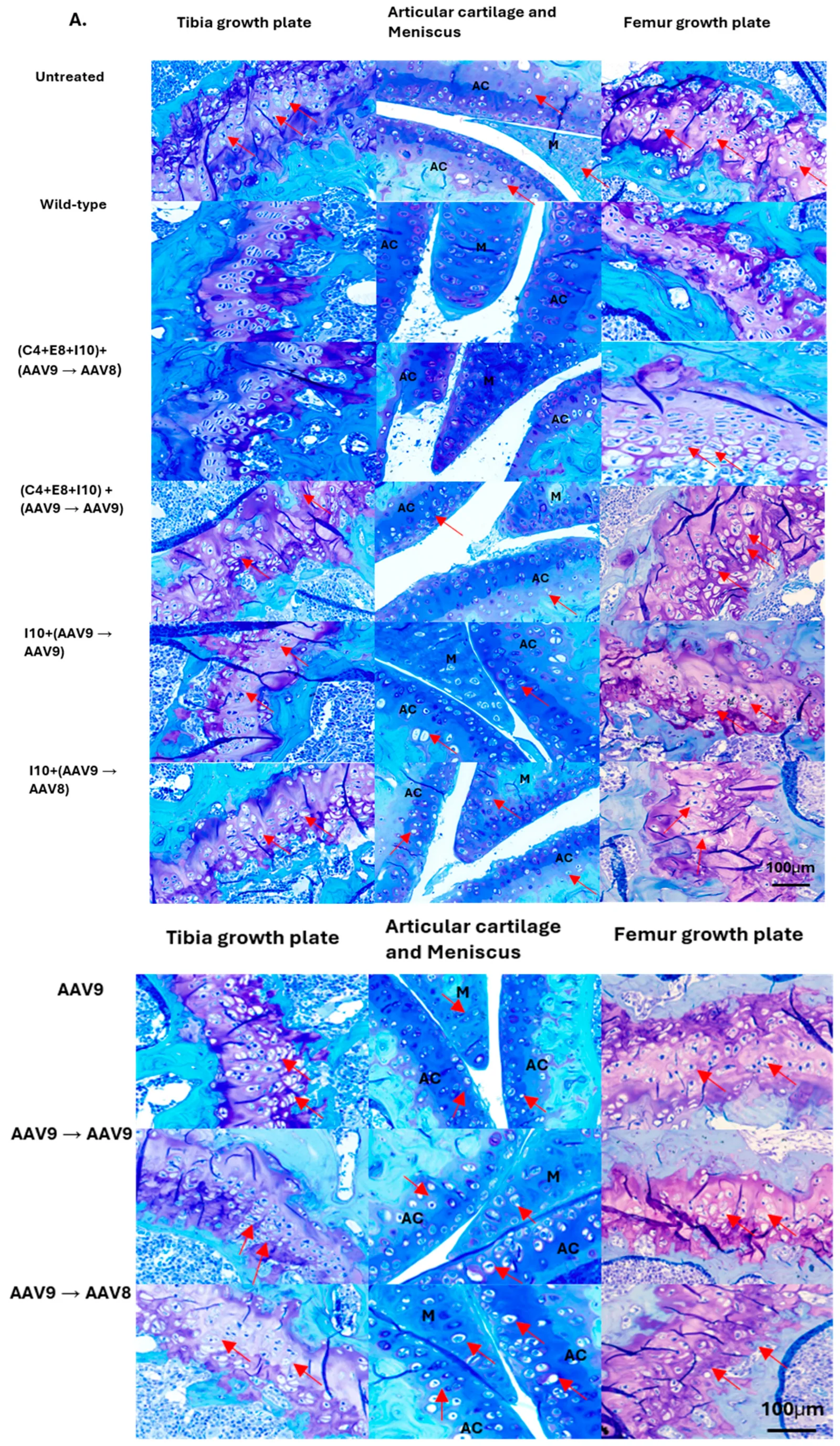

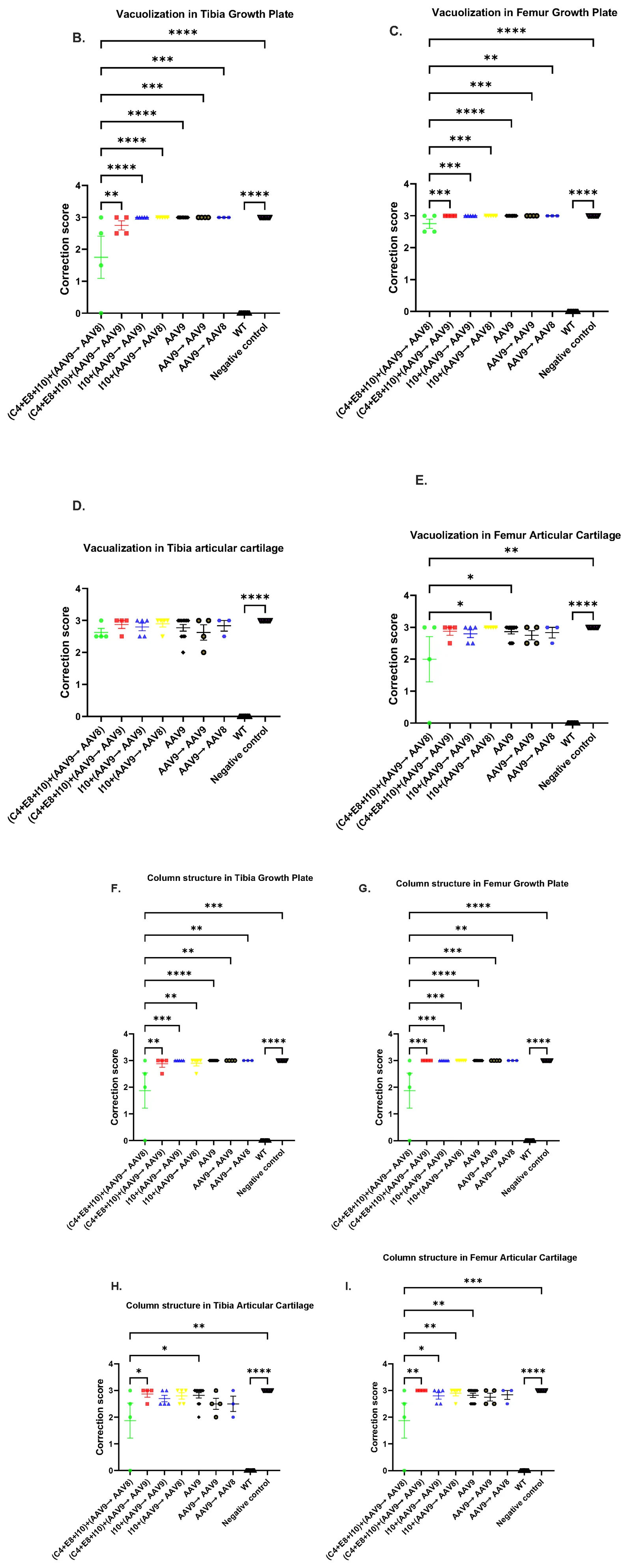

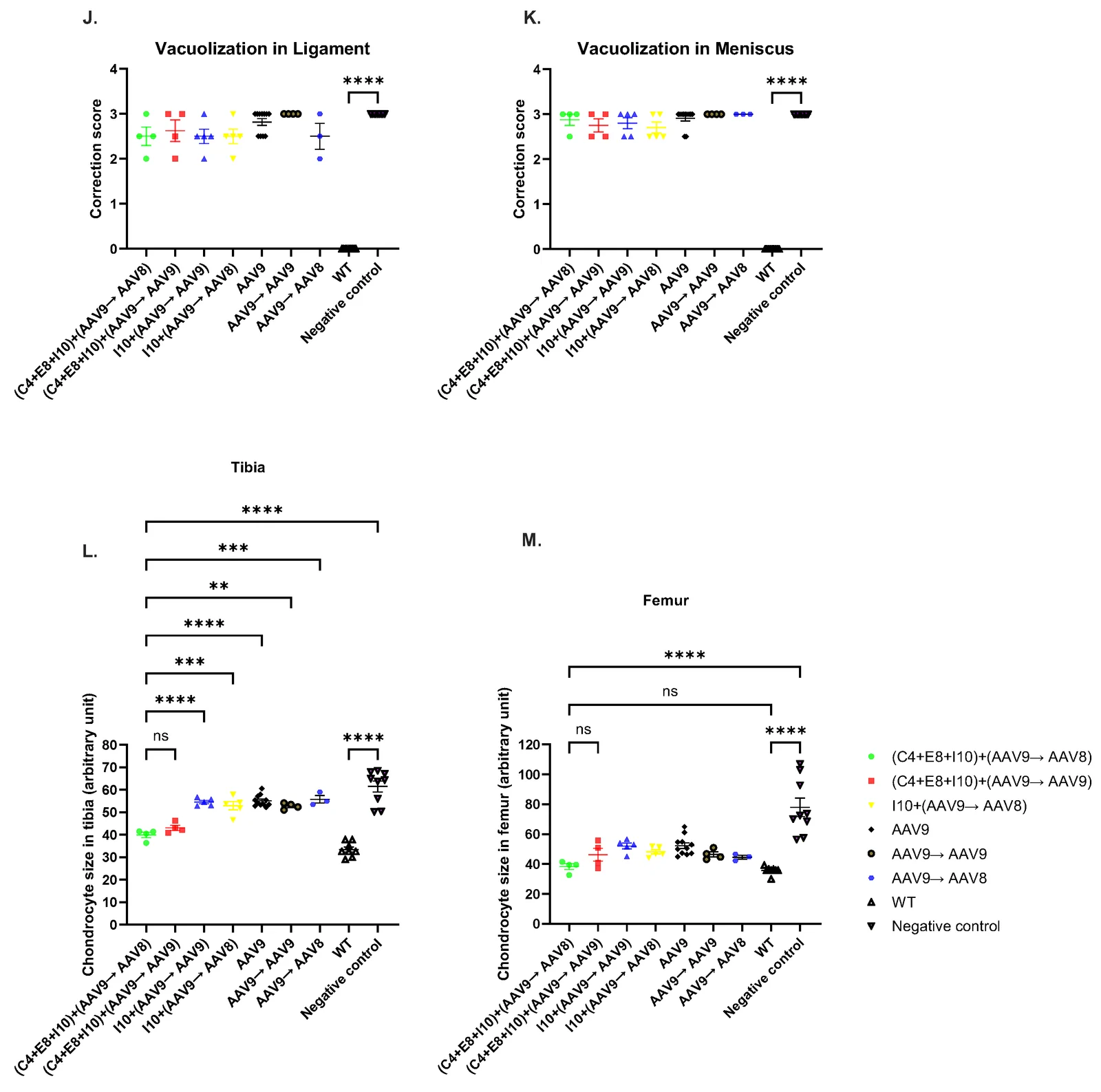
Pathology evaluation of bone. (**A**) Vacuolization in the tibial growth plate, articular cartilage, meniscus, and femoral growth plate at 40× magnification with a 100-µm scale (Red arrow: vacuoles in the chondrocytes, AC = articular cartilage; M = meniscus). (**B**–**E**) Vacuolization in bone. (**F**–**I**) Columnar structure of bone. (**J**,**K**) Vacuolization in the ligament and meniscus, respectively. (**L**) Chondrocyte size in the growth plate regions of XXXXtibia (**M**) Chondrocyte size in the growth plate region of the femur. One-way ANOVA with Tukey’s post hoc test; *: <0.05, **: <0.005, ***: <0.001, ****: <0.0001. *n* = 3–11. Error bars are for the standard error of the mean.

Untreated MPS IVA mice exhibited a disorganized columnar structure in the growth plate and articular cartilage of both the tibia and femur (Figure 7A,F–I; Supplementary Table S2). Although no treated group achieved complete normalization, the [(C4 + E8 + I10) + (AAV9→AAV8)] group showed the greatest improvement in columnar organization, with partial restoration also observed in other treated groups (Figure 7A,F–I; Supplementary Table S2). These findings suggest that combining oral GALNS peptide-induced immunotolerance with dual AAV9 and AAV8 gene therapy yields a slight improvement in bone pathology compared with AAV9-based gene therapy alone.

To determine whether GALNS expression reduced intracellular storage, chondrocyte size was measured in the tibial and femoral growth plates (Figure 7L,M). In the tibia, chondrocyte size remained comparable to untreated MPS IVA mice in all treated groups except [(C4 + E8 + IL-10) + (AAV9→AAV8)] and [(C4 + E8 + IL-10) + (AAV9→AAV9)], which normalized to wild-type levels (Figure 7L). A similar pattern was observed in the femur, in which only these two groups showed that chondrocyte size was restored to wild-type levels (Figure 7M).

**Figure 8:**
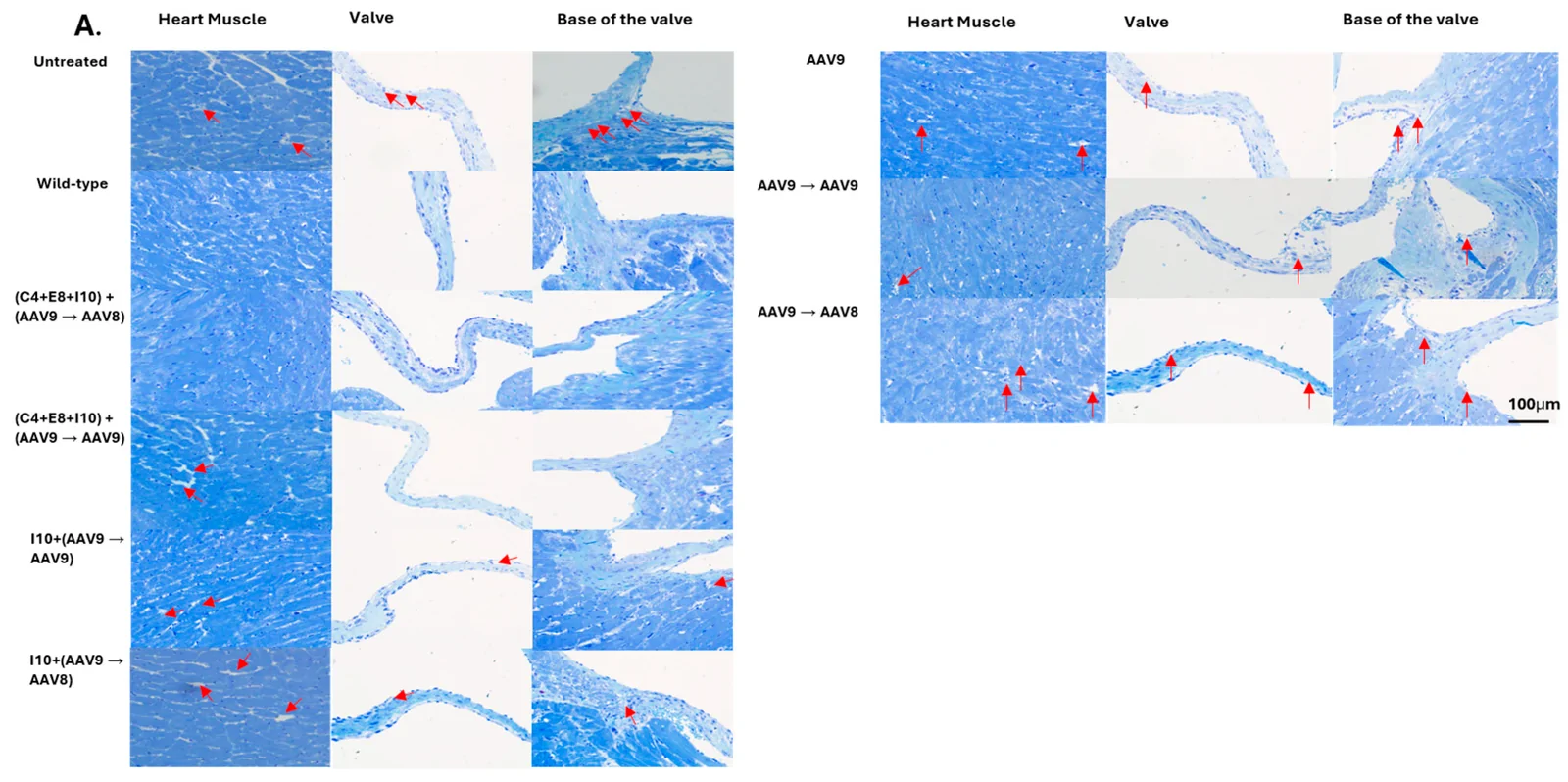

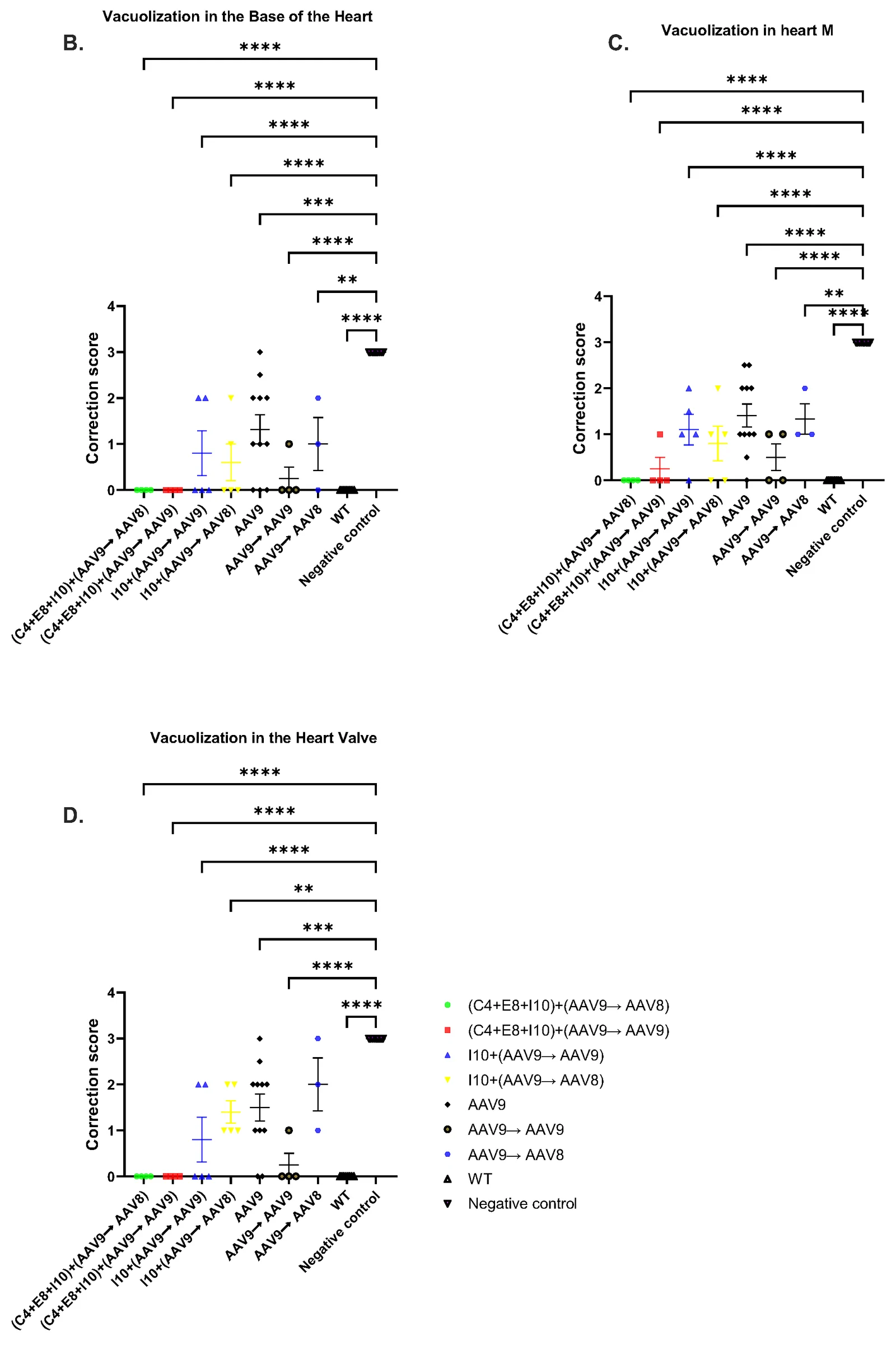
Pathology evaluation of the heart. (**A**) Vacuolization in the base, valve, and muscle of the heart at 40× magnification with a 100-μm scale (Red arrow indicated the vacuoles). Correction score for vacuolization in heart structures; (**B**) Base of the Heart; (**C**) Heart Muscle; (**D**) Heart Valve. One-way ANOVA with Tukey’s post hoc test; **: <0.005, ***: <0.001, ****: <0.0001. *n* = 3–6. Error bars are for the standard error of the mean.

Histopathological evaluation of the heart revealed a significant reduction in vacuolization at the base, valve, and myocardium in all immunotolerant groups compared with the untreated MPS IVA group (Figure 8A–D) (Supplementary Table S3). Notably, the [(C4 + E8 + I10) + (AAV9→AAV8)] group demonstrated complete correction of cardiac pathology across all assessed regions, including the base, valve, and myocardium. In contrast, the non-immunotolerant groups did not show significant improvement in vacuolization at the base of the heart valve. Partial correction was observed in the single-dose AAV9 and dual-dose AAV9 groups, specifically within the heart valve and myocardium, where vacuolization was significantly reduced compared with untreated mice.

### 2.7. Adverse Effect

**Figure 9:**
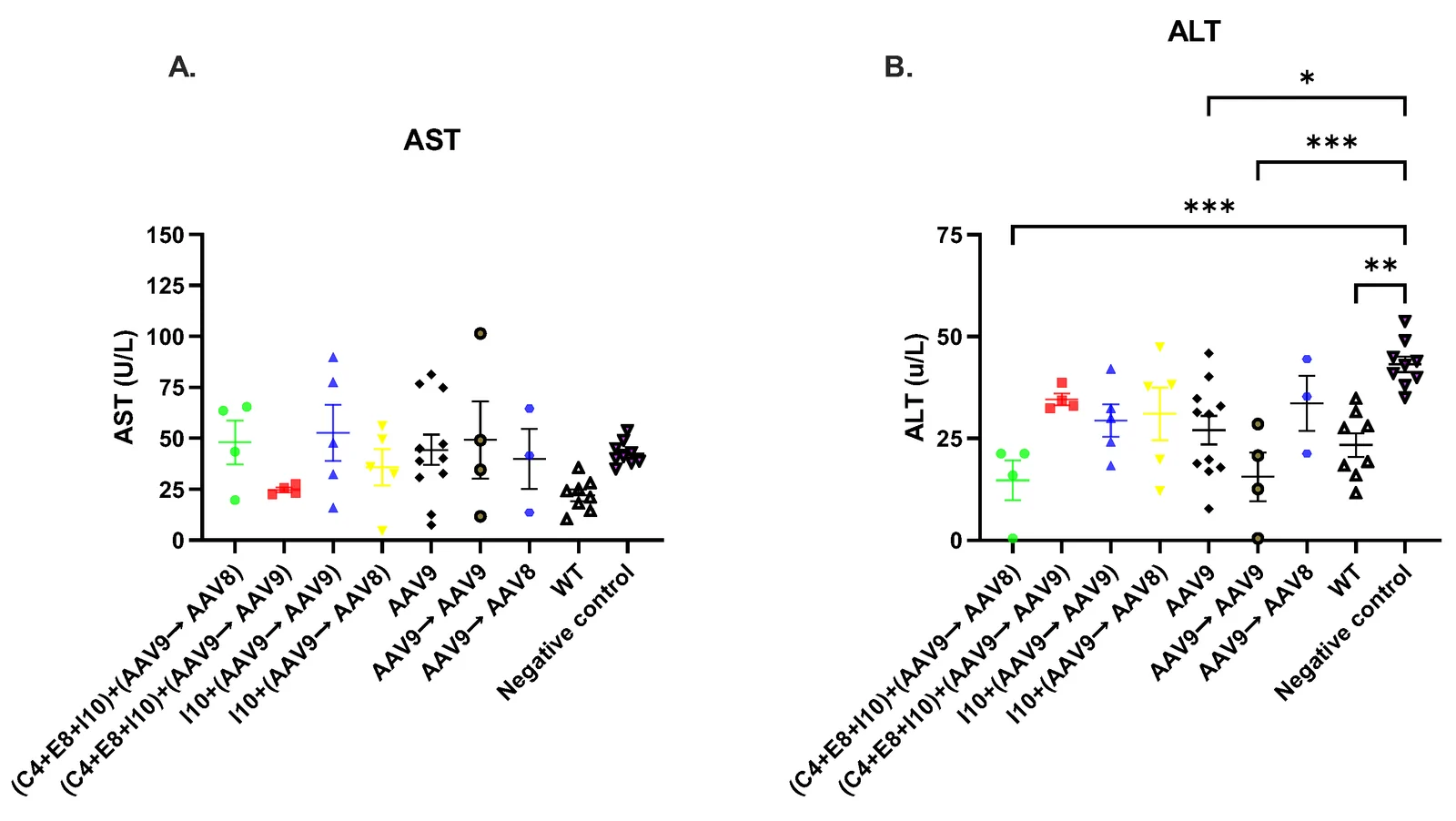
Hepatocellular toxicity analysis. (**A**) AST; (**B**) ALT. One-way ANOVA with Tukey’s post hoc test; *: <0.05, **: <0.005, ***: <0.001, *n* = 3–11. Error bars are for the standard error of the mean.

*a. Liver toxicity.* To evaluate potential liver toxicity associated with AAV-mediated gene therapy, plasma levels of aspartate aminotransferase (AST) and alanine aminotransferase (ALT) were measured at autopsy in 32-week-old mice (Figure 9). AST levels did not differ significantly among treated, untreated MPS IVA, and wild-type groups, remaining within the normal physiological range (46–221 U/mL) (Figure 9A). For ALT, the [(C4 + E8 + I10) + (AAV9→AAV8)] group had the lowest levels compared with all other treated groups and was statistically significant compared with the untreated MPS IVA group. However, ALT levels across all groups remained within the normal range (22–133 U/mL) (Figure 9B). These findings indicate that AAV9 gene therapy did not induce liver toxicity in treated mice.

*b. Body weight (BW).* Body weight (BW) was measured biweekly after AAV9 administration, beginning at 4 weeks post-injection and continuing through 32 weeks of age (Supplementary Figure S1). No significant differences in BW were observed among wild-type, untreated MPS IVA, and treated knockout mice through 16 weeks. Beyond 16 weeks, a significant divergence emerged between wild-type and untreated MPS IVA mice, persisting through study termination at 32 weeks.

Mice receiving a single dose of AAV9 exhibited a gradual increase in BW from 18 to 30 weeks; however, BW remained significantly lower than that of wild-type mice and significantly higher than that of untreated MPS IVA controls. In contrast, mice treated with dual-dose AAV9 or dual-dose AAV9/AAV8 showed a significant reduction in BW relative to wild-type mice after 24 weeks, with values comparable to those of untreated MPS IVA mice. A similar BW trajectory was observed in the I10+ (AAV9→AAV9) and I10+ (AAV9→AAV8) groups.

Notably, the [(C4 + E8 + I10) + (AAV9→AAV8)] and [(C4 + E8 + I10) + (AAV9→AAV9)] groups showed stable BW from 18 weeks onward, maintained through 32 weeks. BW in these groups differed significantly from that of both wild-type and untreated MPS IVA mice (Supplementary Figure S1). These findings suggest that immune tolerance induction during the neonatal period may have activated alternative regulatory mechanisms involving the bone and immune microenvironment, potentially contributing to the observed BW stabilization and reduced age-associated fat accumulation compared with both wild-type and untreated mice. This observation may reflect complex interactions among skeletal development, immune regulation, and systemic metabolism. Given the involvement of skeletal abnormalities in MPS IVA, changes in the bone microenvironment and body composition could potentially influence overall body weight trajectories.

### 2.8. Bone Morphometry by Micro-Computed Tomography

Bone trabecular and cortical structures in the femur were assessed using micro-computed tomography (micro-CT) to evaluate morphometric differences among treated and untreated MPS IVA mice and wild-type controls (Supplementary Figure S2). Compared with wild-type mice, untreated MPS IVA mice showed reductions in bone volume (BV), percent bone volume (BV/TV), and trabecular number (Tb. N), along with an increase in trabecular separation (Tb. Sp), indicating severe disruption of bone architecture. Among the treated groups, [(C4 + E8 + I10) + (AAV9→AAV8)] and I10+ (AAV9→AAV8) groups exhibited slight improvement in BV, BV/TV, and Tb. Sp (Supplementary Figure S2). Moreover, the [(C4 + E8 + I10) + (AAV9→AAV8)], [(C4 + E8 + I10) + (AAV9→AAV9)], and I10 + (AAV9→AAV8) groups demonstrated bone mineral density (BMD) values comparable to those of wild-type mice, reflecting slight restoration of bone morphology. In contrast, the non-immunotolerant groups (AAV9, AAV9→AAV9, and AAV9→AAV8) showed improvement in trabecular separation and cortical thickness but failed to normalize BV, BV/TV, Tb. N, and BMD (Supplementary Figure S2). None of the treated groups differed significantly from the untreated group in other parameters, including total volume of interest, trabecular thickness, degree of anisotropy, cortical thickness, total area, bone area, medullary area, tissue mineral density, or bone length (Supplementary Figure S2).

Overall, the [(C4 + E8 + I10) + (AAV9→AAV8)] group showed a slight improvement in bone morphology, effectively normalizing several key structural parameters, whereas the other treatment groups showed only partial correction.

### 2.9. AAV Genome Biodistribution

Liver samples were used to quantify vector copy numbers per diploid cell using digital PCR (dPCR) [1]. The vector copy number was not detected in the wild-type control and untreated MPS IVA groups (Supplementary Figure S3). All treated groups exhibited high AAV9 vector copy numbers. No significant differences were observed among the treated groups in the liver, lung, ear, and muscle (Supplementary Figure S3).

The lowest vector copy numbers were consistently observed in the liver, ear, lung, and skeletal muscle across all dual-dose AAV9 treatment groups, regardless of whether oral immune modulation was present. This finding suggests induction of anti-AAV9 neutralizing antibodies following the second vector administration. In contrast, animals receiving sequential AAV9 and AAV8 administration demonstrated higher vector copy numbers across these tissues (Supplementary Figure S3).

## 3. Discussion

Antigen-specific oral tolerance to the GALNS transgene enabled effective re-administration of AAV and improved durability in an MPS IVA mouse model. Neonatal exposure to GALNS-derived peptides created a tolerogenic milieu that permitted homologous (AAV9→AAV9) or heterologous (AAV9→AAV8) redosing of 5 × 10^13^ genome copies/kg and yielded higher, more sustained circulating GALNS activity than in non-tolerized animals. The dose was selected based on the preclinical AAV gene therapy experience underlying this MPS IVA model [27] and was intended to provide robust systemic GALNS expression while allowing evaluation of repeat administration. The increase following AAV8 administration demonstrates that the second vector administration resulted in additional GALNS expression. However, the subsequent decrease could reflect several mechanisms, including reduced vector genome persistence, variation in hepatocyte transduction, changes in transgene expression, immune-mediated elimination of transduced cells, or antibody-mediated effects related to the vector capsid. Enzyme activity was inversely correlated with anti-GALNS IgG across groups, indicating reduced antibody-mediated neutralization and improved systemic bioavailability.

Immune profiling supported functional tolerance: peptide-treated mice maintained minimal anti-GALNS IgG and showed IL-10-dominant, IL-2/IL-4-low responses, consistent with regulatory pathway induction and durable transgene expression. IL-2 ELISPOT analysis revealed substantially higher numbers of IL-2-secreting cells in untreated wild-type and MPS IVA control mice than in AAV- and peptide-treated groups following stimulation with GALNS-derived peptides. Although these differences suggest altered cellular cytokine responses among experimental groups, their immunological significance requires cautious interpretation. Because unstimulated splenocyte controls were not included, we could not distinguish GALNS peptide-specific IL-2 secretion from antigen-independent background activity. Consequently, the reduced IL-2 spot counts observed in treated groups cannot independently establish antigen-specific T-cell suppression or immune tolerance.

In non-tolerized cohorts, robust anti-GALNS responses emerged; notably, post-20-week antibody declines did not restore enzyme activity, implying persistent functional inhibition. A single GALNS peptide suppressed detectable anti-GALNS antibodies yet did not increase enzyme activity or efficacy, suggesting a narrow tolerogenic imprint insufficient to sustain transgene bioavailability and cross-correction. In contrast, neonatal multi-peptide exposure likely imprints broader epitope coverage, supporting both immune control and therapeutic benefit [14]. One possible explanation is that simultaneous exposure to multiple GALNS-derived epitopes may broaden the repertoire of antigen-specific regulatory T-cell responses and provide more comprehensive modulation of GALNS-reactive T-cell populations.

At the tissue level, AAV-mediated GALNS expression normalized KS in plasma, liver, and bone. Histopathology showed graded structural improvement: cardiac vacuolization improved in all immunotolerant groups and was fully corrected in the peptide-treated AAV9→AAV8 cohort; skeletal tissues exhibited the greatest recovery. Normalization of chondrocyte size in tibial and femoral growth plates occurred only in immunotolerant dual-dose groups, consistent with a circulating GALNS threshold needed for cross-correction in poorly vascularized cartilage. These data highlight the joint importance of biodistribution, expression level, and threshold-dependent cross-correction for reversing skeletal pathology in MPS IVA [28]. Micro-CT findings corroborated improvements in trabecular/cortical parameters and near-normal bone mineral density in peptide-treated AAV9→AAV8 mice, whereas non-tolerized regimens produced only partial morphometric recovery.

Given constraints on durability from hepatocyte turnover, pediatric liver growth, and subtherapeutic initial dosing, enabling safe, effective redosing is clinically relevant. Rapamycin-loaded nanoparticles (ImmTOR) reduced anti-AAV neutralizing antibodies and permitted redosing in Crigler–Najjar models, but residual low-titer antibodies still attenuated efficacy, indicating a need for complementary strategies [29]. Antigen-specific oral tolerance provides a targeted, non-systemic adjunct that may synergize with capsid-focused approaches. It is important to note that in this study, the oral tolerability strategy used is antigen-specific and targets the therapeutic hGALNS transgene product rather than the AAV capsid. This study suggests that oral administration of GALNS-derived peptides (C4, E8, and I10) may reduce anti-GALNS IgG responses, thereby reducing humoral immunity against the expressed therapeutic enzyme. Contrary to this, antigen-directed immunomodulation strategies, such as ImmTOR-based approaches, aim to suppress immune responses to AAV capsid antigens. AAV gene therapy faces different immunological barriers, so these approaches may complement each other. If GALNS-specific oral tolerance is combined with capsid-targeted immunomodulation, it may reduce immune responses against both the therapeutic transgene product and the viral vector, allowing repeated administration of AAV with greater ease. Nevertheless, the efficacy and safety of such combined strategies remain to be established experimentally.

Serotype switching further mitigates capsid-directed immunity by exploiting limited cross-reactivity among AAV serotypes. Prior work shows that sequential administration of serologically distinct vectors preserves transduction, whereas homologous redosing elevates neutralizing antibodies and blunts expression [30,31,32,33]. Here, switching from AAV9 to AAV8 avoided cross-reactive anti-capsid responses and supported higher tissue vector copy numbers than homologous redosing, which showed evidence of neutralization after the second AAV9 dose. These results support high-dose, repeat-administration strategies as an alternative to single high-dose approaches, with liver enzymes (AST/ALT) remaining within physiological ranges across groups.

Interestingly, mice receiving the combined C4 + E8 + I10 oral peptide regimen followed by sequential AAV9/AAV8 administration exhibited a distinct pattern of body weight stabilization compared with wild-type and untreated MPS IVA mice. This finding may arise from complex interactions between skeletal development, immune regulation and systemic metabolism. Given the significant skeletal involvement in MPS IVA, alterations in the bone microenvironment and body composition could possibly contribute to the observed changes in body weight over time. However, the present study did not directly assess adipose tissue accumulation, metabolic activity, or endocrine function. Therefore, the mechanisms underlying the observed body weight stabilization remain unclear, and the findings should not be interpreted as evidence of improved metabolic regulation or altered fat accumulation. Further studies incorporating longitudinal body composition analysis, metabolic assessments, and endocrine profiling are required to determine whether these factors contribute to the observed phenotype.

Together, oral peptide-induced transgene tolerance and cross-serotype redosing broaden the therapeutic window for MPS IVA gene therapy. Oral tolerance is noninvasive, scalable, and targeted, minimizing off-target immunosuppression while sustaining enzyme availability; serotype switching circumvents capsid-mediated neutralization and preserves transduction upon redosing. This integrated approach yields consistent biochemical and histopathological correction, particularly in cardiac and skeletal tissues, and aligns with existing AAV manufacturing paradigms.

Limitations include species differences in tolerance induction, AAV immunogenicity, and vector persistence, especially beyond the neonatal period. The durability of oral tolerance in older pediatric/adult populations and the feasibility of multiple sequential serotype switches remain uncertain. Divergent late body weight trajectories suggest interactions among immune modulation, skeletal remodeling, and metabolism that warrant mechanistic investigation. Future large-animal work should define optimal timing, dose, and serotype sequence; assess long-term safety (including off-target transduction and integration); and test combinations of antigen-specific tolerance with capsid-directed immunomodulation. These studies are not designed to demonstrate definitive clinical efficacy of immune tolerance induction in humans. Rather, they are intended to establish a proof of concept that antigen-specific immune regulation can be achieved in the setting of GALNS replacement. The mouse model provides a controlled experimental system to define mechanisms of immune tolerance and determine how immune modulation affects therapeutic efficacy. Translation to human studies will require careful identification of appropriate patient populations and may require adjunctive strategies, such as adjuvants that enhance Treg induction [23]. Because human MHC/HLA molecules are highly polymorphic, patient-specific epitope design for OIT may be necessary. To overcome this limitation, we have to consider broader epitope-discovery strategies, including sequence-based AI prediction models and immunopeptidome profiling [34,35], to identify shared GALNS- and AAV-capsid-derived epitopes presented by common HLA alleles. Future studies should include cell-based NAb assays to determine NAbs against vector capsids.

## 4. Materials and Methods

### 4.1. MPS IVA Mouse Model

The MPS IVA knockout mouse (MKC, *Galns^−/−^* KO) on the C57BL/6 background was used as the model for this study [36]. The *Galns^−/−^* KO mouse was generated by targeted deletion of exon 2 of the *Galns* gene [36], resulting in no enzyme activity in the blood and tissues of these mice. Storage materials (GAGs) accumulated in vacuoles of reticuloendothelial Kupffer cells, heart valves, muscles, and chondrocytes, as well as in growth plates and articular cartilage [1]. Wild-type mice served as controls, as they produce physiological levels of the enzyme required for GAG metabolism. All animal care and experiments were reviewed and approved by the Institutional Animal Care and Use Committee of Nemours Children’s Health (Protocol Number: RSP20-12483-003).

### 4.2. Oral Administration with Peptide Epitopes of GALNS

Immunodominant epitopes are distinct components of antigenic regions that are unequivocally recognized by the immune system. A previous study identified highly immunogenic regions of the GALNS enzyme in C57BL/6 (H2-IAb) using algorithms to predict B- and T-cell epitopes [36]. The study aimed to create a detailed profile of the most immunogenic regions in the GALNS enzyme to predict B- and T-cell epitopes. Ten 20-mer peptides encompassing immunogenic regions in the GALNS enzyme were evaluated, and the C4, E8, and I10 peptides were found to be the most immunogenic (Table S1). Male mice were orally administered a mixture of the three peptides (C4 + E8 + I10) or a single peptide (I10) within 72 h of birth every other day for 20 days at doses of 3 µg/g BW. Postnatal day 3 (PND3) was selected based on our previous GALNS oral-tolerance study [23] and should not be interpreted as demonstrating that PND3 is the optimal tolerance window. The mixture of peptides contained equal amounts of C4, I10, and E8 peptides (Table S1). The experimental design is shown in Figure 1, with nine experimental groups (*n* = 3–11): (1) MPS IVA mice orally administered a mixture of the three peptides (C4 + E8 + I10) at 3 µg/g BW followed by AAV9 and AAV8 at two different time points [(C4 + E8 + I10) + (AAV9→AAV8)]; (2) MPS IVA mice orally administered a mixture of the three peptides (C4 + E8 + I10) at 3 µg/g BW, followed by dual AAV9 at two different time points [(C4 + E8 + I10) + (AAV9→AAV9)]; (3) MPS IVA mice orally administered a single peptide (I10) at 3 µg/g BW, followed by dual AAV9 at two different time points [I10) + (AAV9→AAV9)]; (4) MPS IVA mice orally administered a single peptide (I10) at 3 µg/g BW followed by AAV9 and AAV8 at two different time points [I10 + (AAV9→AAV8)]; (5) MPS IVA mice orally administered PBS followed by a single AAV9 injection (AAV9); (6) MPS IVA mice orally administered PBS followed by dual AAV9 at two different time points (AAV9→AAV9); (7) MPS IVA mice orally administered PBS followed by AAV9 and AAV8 at two different time points (AAV9→AAV8); (8) Untreated wild-type mice (WT); (9) Untreated MPS IVA mice (negative control).

### 4.3. AAV Gene Therapy for Mouse Models

As shown in Supplementary Figure S3A, the AAV expression cassette was constructed with an optimized *GALNS* coding sequence downstream of a ubiquitous Cytomegalovirus Enhancer/Chicken β-Actin (CAG) promoter, which consists of a modified chicken β-actin promoter linked to a cytomegalovirus early enhancer element [23]. The expression cassette consists of the *GALNS* coding sequence followed by the Woodchuck hepatitis virus Post-transcriptional Regulatory Element (WPRE) and a bovine growth hormone polyadenylation signal. The viral genome was packaged into AAV9 and AAV8 vectors by Vector Builder and named pAAV9 [Exp]-CAG> {hGALNSco}: WPRE and pAAV8 [Exp]-CAG> {hGALNSco}: WPRE, respectively (Supplementary Figure S3A). The recombinant AAV9 and AAV8 vectors contained a codon-optimized human GALNS (hGALNS) transgene under the control of the CAG regulatory element, comprising the cytomegalovirus (CMV) immediate-early enhancer, chicken β-actin promoter, and a chimeric chicken β-actin/rabbit β-globin intron. The expression cassette additionally contained the woodchuck hepatitis virus post-transcriptional regulatory element (WPRE) and bovine growth hormone polyadenylation signal.

5 × 10^13^ genome copies (GC)/kg of AAV9 vectors were administered via the tail vein to mice at 4 weeks of age in the previously mentioned groups 1–8 (Figure 1). A second intravenous injection of the same dose of AAV9 or AAV8 was administered to mice that had previously received AAV9 at 10 weeks of age, except for group 5 (Figure 1).

### 4.4. Anti-GALNS and Anti-AAV Antibodies by ELISA

As reported previously, ELISA (Enzyme-Linked Immunosorbent Assay) was used to quantify anti-GALNS antibodies in plasma samples [1]. A 96-well microtiter plate was coated with 2 g/mL of the purified GALNS enzyme (elosulfase alfa) in 15 mM Na_2_CO_3_, 35 mM NaHCO_3_, and 0.02% NaN_3_ (pH 9.6) per well overnight. The plate was blocked with 3% bovine serum albumin in PBS (pH 7.2) for 1 h at room temperature, followed by three washes with Tris-buffered saline with Tween 20 (TBS-T) (10 mM Tris [pH 7.5], 150 mM NaCl, 0.05% Tween 20). After two more TBS-T washes, 100-fold-diluted mouse plasma was added to each well, and the wells were incubated for an additional 2.5 h at 37 °C. Peroxidase-conjugated goat anti-mouse immunoglobulin G (IgG) (Thermo Fisher Scientific) was added to the plate at a 1:1000 dilution after four washes with TBS-T. The plate was then incubated for an additional hour at room temperature. In total, 100 μL of peroxidase substrate (ABTS solution, Invitrogen, Carlsbad, CA, USA) was added to each well after three TBS-T washes, followed by two TBS washes (10 mM Tris, pH 7.5, 150 mM NaCl). The plate was then incubated for 30 min at room temperature. After stopping the reaction with 1% SDS, the absorbance at 410 nm was measured in optical density units using a PerkinElmer Victor X4 plate reader (PerkinElmer, Waltham, MA, USA). The standard curve was generated using repeated dilutions of known monoclonal anti-mouse IgG concentrations added to the GALNS-coated plate. The optical density was converted to μg of IgG per milliliter of plasma using linear regression analysis. The same ELISA protocol was used to detect anti-AAV antibodies, except that 96-well plates were coated with AAV9 or AAV8 vectors.

### 4.5. Detection of Pro- and Anti-Inflammatory Cytokines Using ELISPOT

The ELISPOT assay was used to detect cytokine (IL-2, IL-4, and IL-10) secretion by activating immune cells according to the manufacturer’s protocol (ImmunoSpot, Cleveland, OH, USA). The assay was performed in 96-well plates with nitrocellulose membranes coated with specific primary antibodies for IL-2, IL-4, and IL-10. Single-cell suspensions of splenocytes (1 × 10^5^ cells/well), combined with an appropriate stimulus (10 µg/mL of cells, peptide mixture, Table S1), were added to the wells of the ELISPOT plate after blocking for 24 to 48 h. After removing the cells (1 × 10^5^ cells/well) and washing, biotinylated secondary detection antibodies, enzyme-labeled streptavidin conjugates, and chromogenic substrate solution were added stepwise to form colored spots. The number of spots formed was enumerated automatically using the ImmunoSpot reader (C.T.L. A VION Biosciences Company, Cleveland, OH, USA).

### 4.6. GALNS Enzyme Activity Assay

GALNS enzyme activities in plasma and various tissues were determined as described previously [1]. A Bead Mill Homogenizer (OMNI International, Kennesaw, GA, USA) was used to homogenize frozen tissues in a solution containing 25 mmol/L Tris-HCl (pH 7.2) and 1 mmol/L phenylmethylsulfonyl fluoride. After transferring the homogenates to microcentrifuge tubes, they were centrifuged at 4 °C for 30 min at 12,000 rpm. The supernatants were collected. Accordingly, 9 μL of the GALNS substrate containing 22 mM 4-methylumbelliferyl-β-galactopyranoside-6-sulfate (Biosynth, Gardner, MA, USA) prepared in 0.1 M NaCl/0.1 M sodium acetate (pH 4.3) was incubated with 2 μL of tissue lysate or plasma at 37 °C for 16 h. After this first incubation, 2 µL of 10 mg/mL Aspergillus oryzae β-galactosidase (Sigma-Aldrich, St. Louis, MO, USA) in 0.1 M NaCl/0.1 M sodium acetate (pH 4.3) was added to break down the enzymatic reaction product, 4-methylumbelliferyl-β-D-galactosidase (MUG), and the mixture was incubated for an additional two hours at 37 °C to release 4-methylumbelliferone (4-MU) as a fluorescent product. After stopping the reaction with a 1 M glycine-NaOH (pH 10.5) solution, a PerkinElmer Victor X4 plate reader (PerkinElmer, Waltham, MA, USA) was used to read the fluorescence signal of 4-MU at excitation 366 nm and emission 450 nm. Enzyme activity was expressed as nanomoles of 4-methylumbelliferone produced per hour per microliter of plasma or per milligram of tissue protein. The total protein concentration was measured using a Bicinchoninic Acid (BCA) protein assay kit (Thermo Fisher Scientific, Waltham, MA, USA).

### 4.7. Glycosaminoglycan Quantification in Plasma and Tissues

Extraction of GAG from different tissues (liver and humerus) was performed using the previously described protocol [2]. Acetone was used as a defatting solvent to homogenize frozen tissue samples. To extract GAG chains from core proteins, the homogenate was centrifuged at 4 °C for 30 min, and the pellet was then dried by vacuum centrifugation. The pellet was suspended in 0.5 M NaOH and incubated at 50 °C for 2 h. The solution was neutralized with 1 M HCl, then diluted with 3 M NaCl. The supernatant was recovered after centrifugation to remove nucleotides and to adjust the pH to below 1.0 with an additional 1 M HCl. After further centrifugation, the precipitated proteins were removed from the supernatant, and 1 M NaOH was added to neutralize the supernatant. The supernatant was mixed with two volumes of ethanol containing 1.3% potassium acetate and centrifuged for 30 min at 4 °C to precipitate the crude GAGs. Following centrifugation, the GAGs were dried and purified with 80% ethanol, then reconstituted in 50 mM Tris-HCl, pH 7.0, and stored at −20 °C. As previously described, liquid chromatography-tandem mass spectrometry (LC-MS/MS) was employed to quantify KS in plasma and tissues [2].

### 4.8. Pathological Assessment by Toluidine Blue Staining

Toluidine blue was used to stain heart and knee joint samples from mice, as described previously [1]. Heart and knee joints were collected from 32-week-old treated and untreated MPS IVA and wild-type mice at autopsy in 10% formalin. Light microscopy was used to evaluate lysosomal storage in vacuoles of chondrocytes and cardiovascular tissues after fixation in 2% paraformaldehyde and 4% glutaraldehyde, followed by toluidine blue staining of 0.5 µm thick sections. Pathological slides of knee joints and hearts were scored for vacuolization and column structure in a double-blind manner to minimize bias. Each slide was scored from 0 (no visible vacuolization) to 3 (obvious vacuolization) for column structure and vacuolization in the growth plate and articular cartilage [1]. Similar scoring for vacuolization was applied to the heart muscle, base, and valve. Statistical analysis was performed on all heart and knee-joint scores to determine the reduction in storage materials.

### 4.9. Toxicity Analysis

We measured alanine aminotransferase (EALT-100, BioAssay Systems, Hayward, CA, USA) and aspartate aminotransferase (EASTR-100, BioAssay Systems) levels in plasma from all treated and untreated mice at 16 weeks of age, following the manufacturer’s instructions, to assess AAV-GT toxicity. All analyses were conducted on 96-well plates and read at the wavelength provided by the supplier using a PerkinElmer Victor X4 plate reader (PerkinElmer, Waltham, MA, USA).

### 4.10. Plasma and Tissue Collection

From 4 to 32 weeks, blood was drawn biweekly from all mouse groups using a 22G needle to puncture the superficial temporal vein into EDTA-containing tubes (BD Microtainer). After centrifugation at 10,000 rpm for 10 min, the plasma was collected and stored at −20 °C. Following RBC lysis with 0.2% NaCl *w*/*v*, WBCs were extracted from the red blood cells. At 32 weeks of age, all mice were euthanized in a CO_2_ chamber and perfused with 20 mL of 0.9% normal saline. For further testing of KS and GALNS activity, the liver, kidney, lung, spleen, heart, trachea, femur, tibia, and knee joint were collected on dry ice and stored at −80 °C. In addition, other tissues, including bone and heart, were collected and fixed in 10% neutral-buffered formalin for histopathological analysis. The tibia was stored in 100% ethanol for the micro-CT assay.

### 4.11. Micro-CT

Micro-CT analysis of the femur was performed to characterize trabecular and cortical structure and morphometry, as described previously [36]. Briefly, femurs were collected at autopsies from 32-week-old MPS IVA and wild-type mice and fixed in 100% ethanol. For scanning, the samples were wrapped in gauze, soaked in 0.9% NaCl, and scanned on a SkyScan 1276 micro-CT system (Bruker, Billerica, MA, USA).

### 4.12. AAV Vector Genome Biodistribution

An AAV vector containing expression cassettes for the human GALNS transgene, driven by the CAG promoter, was used in this study (Supplementary Figure S3A). The number of vector copies in the liver was quantified using digital PCR, as previously described [37]. Briefly, liver samples were used to extract DNA using the Gentra Puregene kit according to the manufacturer’s instructions (QIAGEN, Germantown, MD, USA). Genomic DNA from frozen liver samples of mice was analyzed by digital PCR (dPCR) using specific primers and probes: 5′-AGAAGCCCTGAGCAGAATCA-3′, 5′-CAGTTCATCACGGCCCAATTAC-3′, and 5′-AGCAGCACCAAGAGGCTCTGGTTC-3′.

### 4.13. Statistical Analysis

All statistical analyses were conducted using GraphPad Prism 9.50 (GraphPad, San Diego, CA, USA). We conducted Shapiro–Wilk and Kolmogorov–Smirnov tests to determine whether the data followed a normal distribution. For normally distributed quantitative data, we calculated the mean and standard error; for non-normally distributed quantitative data, we calculated the median and 95% confidence interval. To analyze more than two groups when the distributions were normal, we used one-way analysis of variance (ANOVA) with Tukey’s post hoc test or the Kruskal–Wallis test with Dunn’s multiple-comparison tests [28].

## 5. Conclusions

In summary, selective transgene-specific tolerance, paired with serotype switch redosing, offers a generalizable, clinically actionable framework for repeated AAV administration in pediatric enzyme deficiency disorders constrained by immune barriers and tissue turnover, and supports translational advancement for Morquio A syndrome.

## Data Availability

All data are available upon request.

## Supplementary Materials

The following supporting information can be downloaded at https://www.mdpi.com/article/10.3390/ijms27219553/s1.
