# Supplementary material for: Induction of Oral Immune Tolerance Enables Sequential AAV9/AAV8 Gene Therapy in Mucopolysaccharidosis IVA
Source: Int J Mol Sci. 2026 Oct 26;27(21):9553. doi: 10.3390/ijms27219553 (PMC13762537; doi:10.3390/ijms27219553)
Supplement: Supplementary file 1 [file ijms-27-09553-s001.zip › ijms-4605890-supplementary.pdf]

Oral Immune Tolerance Induction Enables Sequential AAV9/AAV8 Gene Therapy for Mucopolysaccharidosis IVA

Sampurna Saikia[1,2], Krishna Sai Musini[2], Shaukat Khan[2], Shunji Tomatsu[1,2,3,4,5],

Supplementary Data

Table S1: Peptide sequences chosen epitopes of GALNS[1].

Peptide No.	GALNs region	Structural Domain	Sequence	Type of Epitope
C4	135-154	Loop	PNCHFGPYDNKARPNIPVYR	B/T
E8	200-219	β-sheet/loop	FFLYWAVDATHAPVYASKPF	T
I10	447-466	α-helix/loop	QQHQEALVPAQPQLNVTNWA	T

Table S2: Bone pathology at 32 weeks. Levels of storage materials and degrees of disoriented columns were 2 (++), and "marked" was 3 (+++). Each pathological slide was assessed in a double – blind manner three times, n=4-8. Data are presented as Mean±. SD

**Vacuolization**	**UT**	**WT**	**(C4+E8+I10) + (AAV9⟶AAV8)**	**(C4+E8+I10) + (AAV9⟶AAV9)**	**I10+ (AAV9⟶AAV9)**	**I10+ (AAV9⟶AAV8)**	**AAV9**	**AAV9⟶AAV9**	**AAV9⟶AAV8**
Tibia Growth Plate	3±0	0±0	2.45±0.896	2.88±0.211	3±0	3±0	2.97±0.114	3±0	3±0
Tibia Articular Cartilage	3±0	0±0	2.5±0.852	2.93±0.176	2.76±0.259	2.85±0.241	2.83±0.297	2.81±0.372	2.75±0.223
Femur Growth Plate	3±0	0±0	2.5±0.968	3±0	3±0	3±0	3±0	3±0	3±0
Femur Articular Cartilage	3±0	0±0	2.55±1.013	2.83±0.25	2.76±0.258	3±0	2.82±0.243	2.66±0.258	2.83±0.258
Ligament	3±0	0±0	2.57±0.449	2.6±0.418	2.5±0.267	2.5±0.408	2.77±0.263	3±0	2.25±0.353
Meniscus	3±0	0±0	2.85±0.243	2.7±0.273	2.81±0.258	2.87±0.256	2.94±0.166	3±0	3±0
**Column structure**									
Tibia Growth Plate	3±0	0±0	2.41±0.86	2.9±0.166	2.96±0.125	2.95±0.158	2.95±0.208	2.9±0.189	2.91±0.204
Tibia Articular Cartilage	3±0	0±0	2.45±0.879	2.93±0.176	2.77±0.261	2.81±0.252	2.81±0.228	2.64±0.378	2.57±0.449
Femur Growth Plate	3±0	0±0	2.54±0.907	3±0	3±0	3±0	3±0	3±0	2.41±0.223
Femur Articular Cartilage	3±0	0±0	2.44±0.982	2.87±0.353	2.83±0.244	2.81±0.258	2.78±0.253	2.60±0.223	2.75±0.273

Table S3: Heart pathology at 32 weeks. Levels of storage materials and degrees of disoriented columns were 2 (++), and "marked" was 3 (+++). Each pathological slide was assessed in a double – blind manner three times, n=4-8. Data are presented as Mean±. SD

Vacuolization	UT	WT	(C4+E8+I10)+ (AAV9⟶AAV8)	(C4+E8+I10)+ (AAV9⟶AAV9)	I10+ (AAV9⟶AAV9)	I10+ (AAV9⟶AAV8)	AAV9	AAV9⟶AAV9	AAV9⟶AAV8
Base	3±0	0±0	0±0	0±0	0.57±0.975	0.5±0.755	0.92±1.03	0.14±0.377	1±0.816
valve	3±0	0±0	0±0	0±0	0.57±0.975	0.375±0.517	1.02±1.07	0.14±0.377	1.5±1.29
Muscle	3±0	0±0	0±0	0.25±0.5	0.78±0.809	0.5±0.755	1.21±0.767	.428±0.534	1±0.816

Figure S1: Biweekly measurement of body weight; Two-way ANOVA with Tukey's post hoc test for plasma week-by-week; p-values expressed as: *: < 0.05, **: < 0.005,**: < 0.005,***: < 0.001,****: < 0.0001. n = 3-11. Error bars are for the standard error of the mean

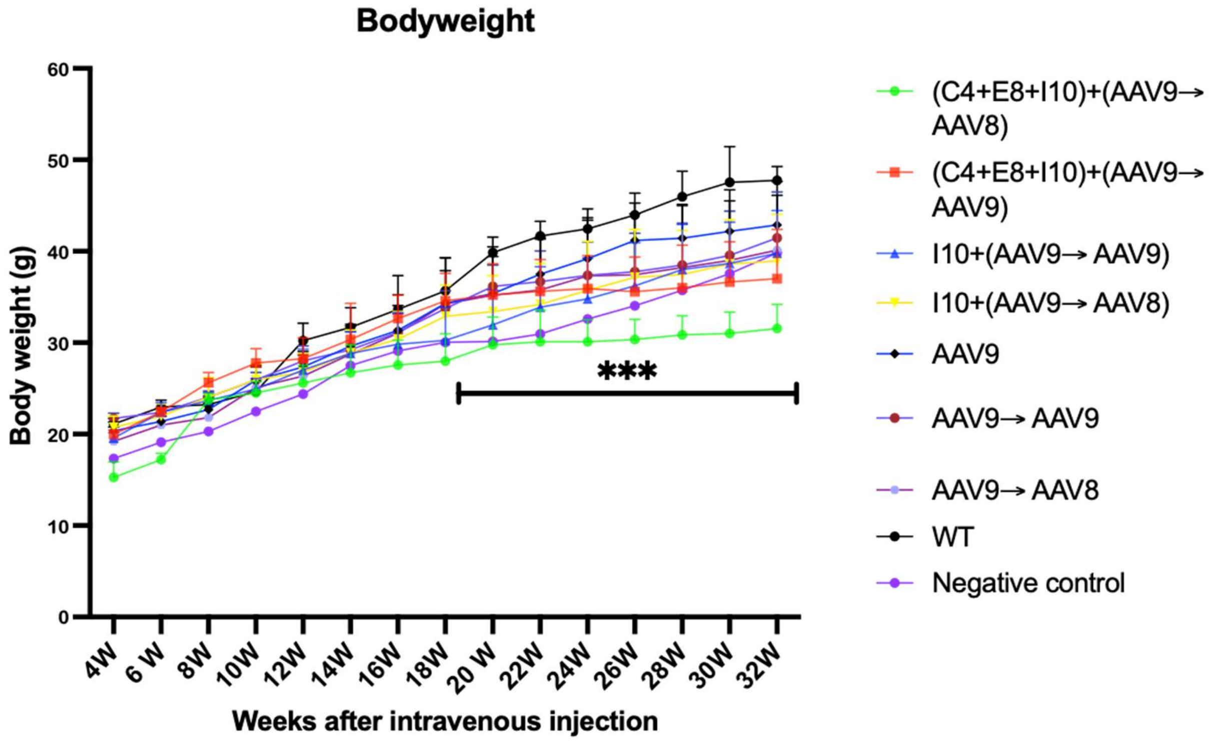

Figure S2: Bone morphometric analysis. Trabecular and Cortical bone morphology (1-8). One-way ANOVA with Tukey's post hoc test; *: < 0.05, **: < 0.005, ***: < 0.001, ****: < 0.0001. n = 3-11. Error bars are for the standard error of the mean.

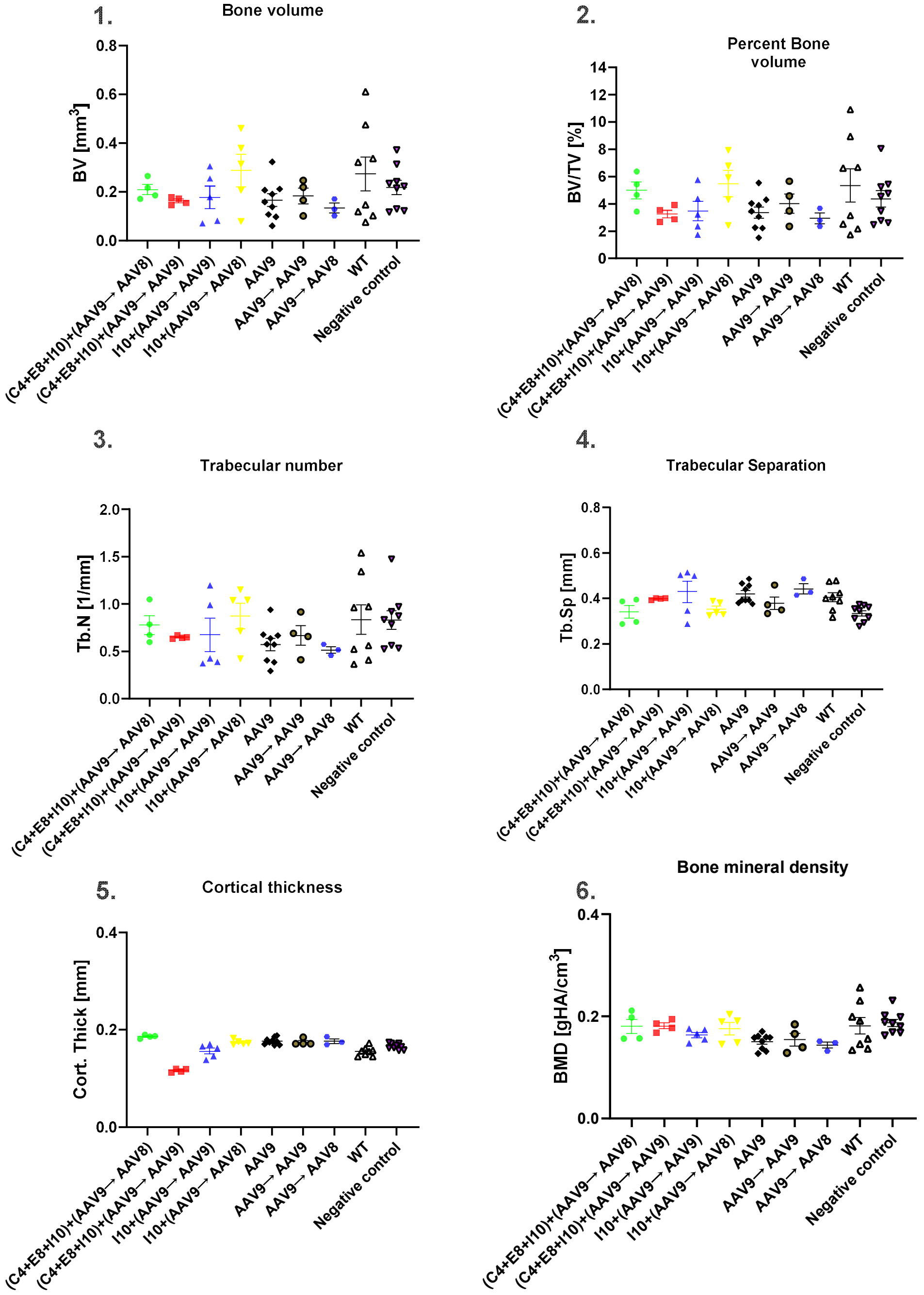

7.

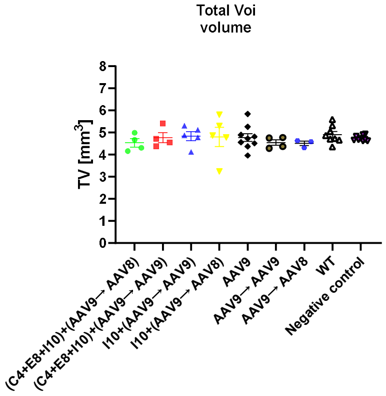

8.

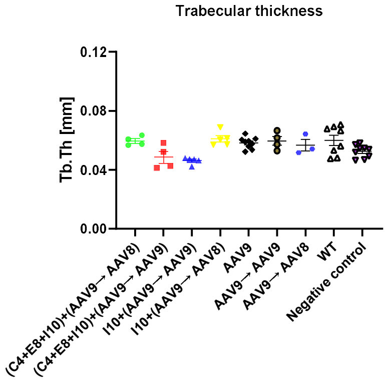

9.

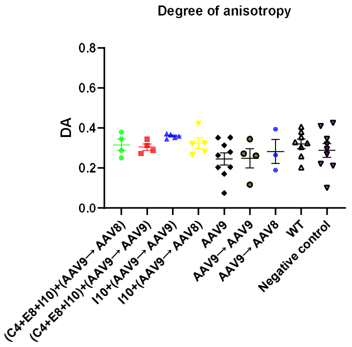

10.

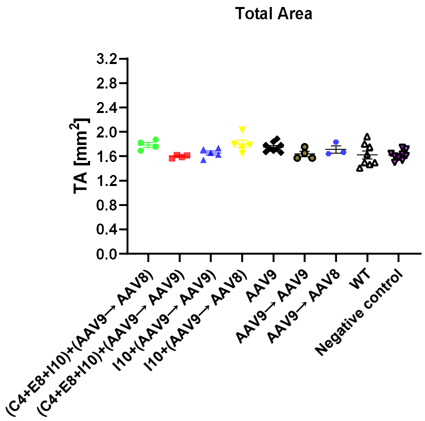

11.

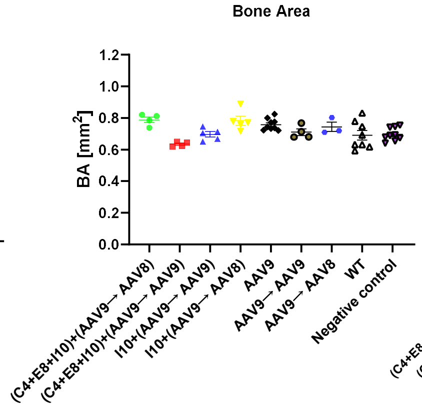

12.

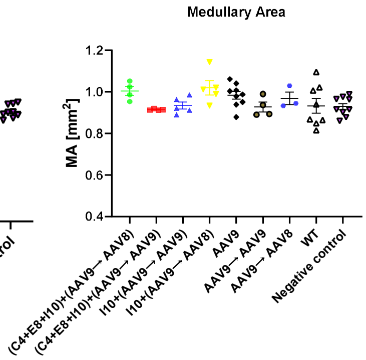

13.

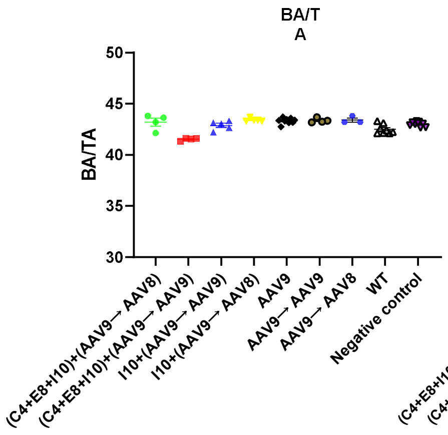

14.

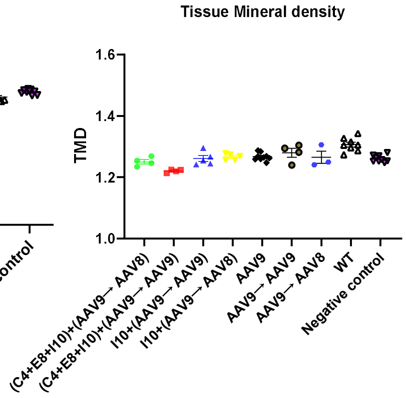

15.

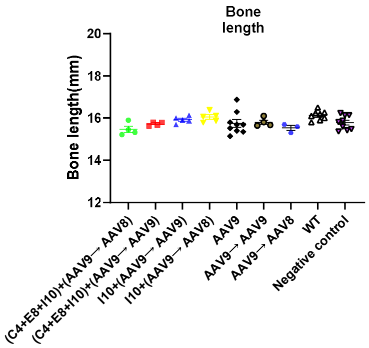

Figure S3: A. Vector genome construct; B. Vector genomic copies per diploid genome in 1. liver;2. Ear; 3. Lung; 4. Muscle One-way ANOVA with Tukey's post hoc test; *: < 0.05**: < 0.005, **: < 0.005,***: < 0.001,****: < 0.0001., n = 3-11. Error bars are for the standard error of the mean.

A.

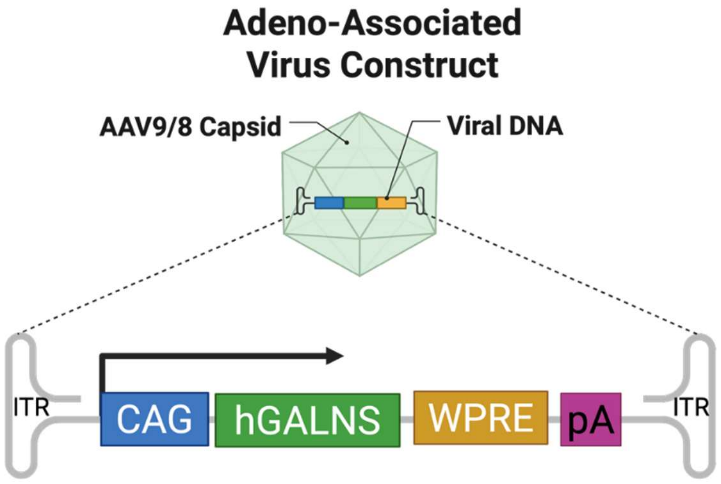

B.

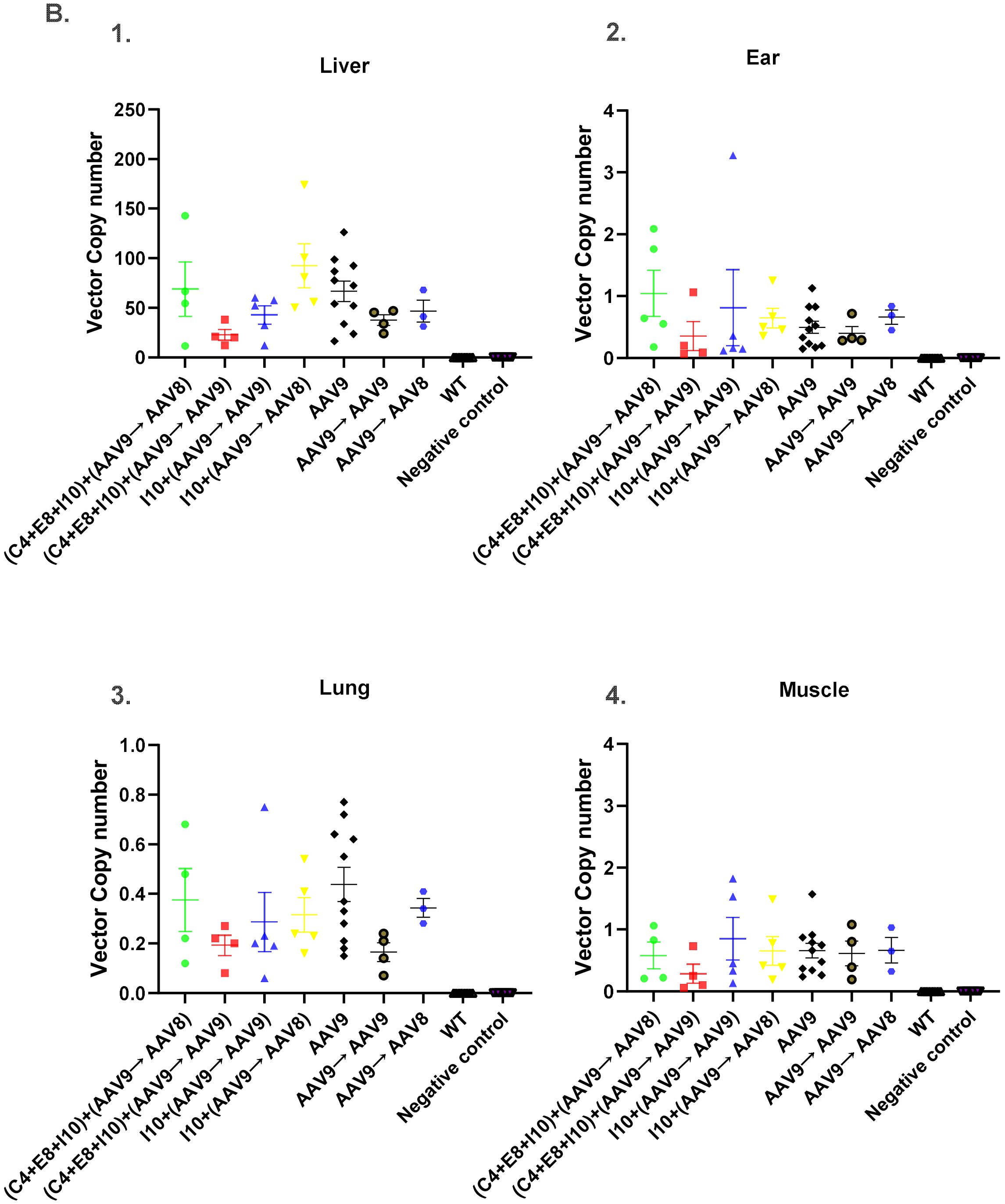
1.
Liver
Vector Copy number
250
200
150
100
50
0
(C4+E8+I10)+(AAV9→ AAV8)
(C4+E8+I10)+(AAV9→ AAV9)
I10+(AAV9→ AAV9)
I10+(AAV9→ AAV8)
AAV9
AAV9→ AAV9
AAV9→ AAV8
WT
Negative control
2.
Ear
4
3
2
1
0
3.
Lung
1.0
0.8
0.6
0.4
0.2
0.0
4.
Muscle

Reference:

1. Sosa, A.C., Kariuki, B., Gan, Q., et al. (2020). Oral immunotherapy tolerizes mice to enzyme replacement therapy for Morquio A syndrome. *J. Clin. Invest.* 130, 1288–1300.
